# Constructing Intrinsically Safe Lithium-Ion Battery Energy Storage via Gradient-Laminated Ceramifiable Silicone Foams

**DOI:** 10.1007/s40820-026-02228-2

**Published:** 2026-05-24

**Authors:** Shuilai Qiu, Jingyao Xu, Congling Shi, Laibin Zhang

**Affiliations:** 1 College of Safety and Ocean Engineering, China University of Petroleum-Beijing, 18 Fuxue Road, Beijing, 102249 People’s Republic of China; 2 Key Laboratory of Oil and Gas Safety and Emergency Technology, Ministry of Emergency Management, Beijing, 102249 People’s Republic of China; 3 Beijing Key Laboratory of Metro Fire and Passenger Transportation Safety, China Academy of Safety Science and Technology, 32 Beiyuan Road, Beijing, 100012 People’s Republic of China

**Keywords:** Thermal runaway propagation, Gradient-laminated structure, Ceramifiable silicone foam, Intrinsically safe, Energy storage systems

## Abstract

Achieving high safety in energy storage systems is paramount but hindered by the catastrophic risks of thermal runaway propagation (TRP). This study develops a gradient-laminated ceramifiable silicone foam composite to resolve the inherent trade-off between thermal insulation and dynamic impact toughness. By integrating a polydimethylsiloxane foam matrix with a load-bearing glass fiber fabric skeleton, the material utilizes silane coupling agents for robust interfacial adhesion, while multiscale fillers promote synergistic ceramicization. Characterization reveals robust mechanical durability, maintaining stable elasticity across a wide temperature range (− 40 to 300 °C) and retaining 93% residual stress after 1,000 compression cycles. Under extreme thermal exposure, the foam transforms into a dense ceramic barrier, reducing total heat release by 54.4% and sustaining thermal protection for over 30 min. Crucially, during battery module testing, this architecture efficiently intercepts high-velocity gas jets and confines thermal runaway to a single cell. Fabricated via a scalable process, this composite paves a viable way for constructing intrinsically safe energy storage systems.

**Supplementary Information:**

The online version contains supplementary material available at 10.1007/s40820-026-02228-2.

## Highlights

- A gradient laminated architecture is engineered to resolve the intrinsic trade off between thermal insulation and impact toughness via synergistic ceramization.
- The composite exhibits exceptional fatigue resistance with 93% stress retention and stable elasticity across a wid e temperature range ( from 40 to 300 °C).
- The formed dense ceramic barrier effectively intercepts high pressure gas jets, confining thermal runaway to a single cell in battery modules.

**Supplementary Information:**

The online version contains supplementary material available at 10.1007/s40820-026-02228-2.

## Graphical abstract

**Figure:**
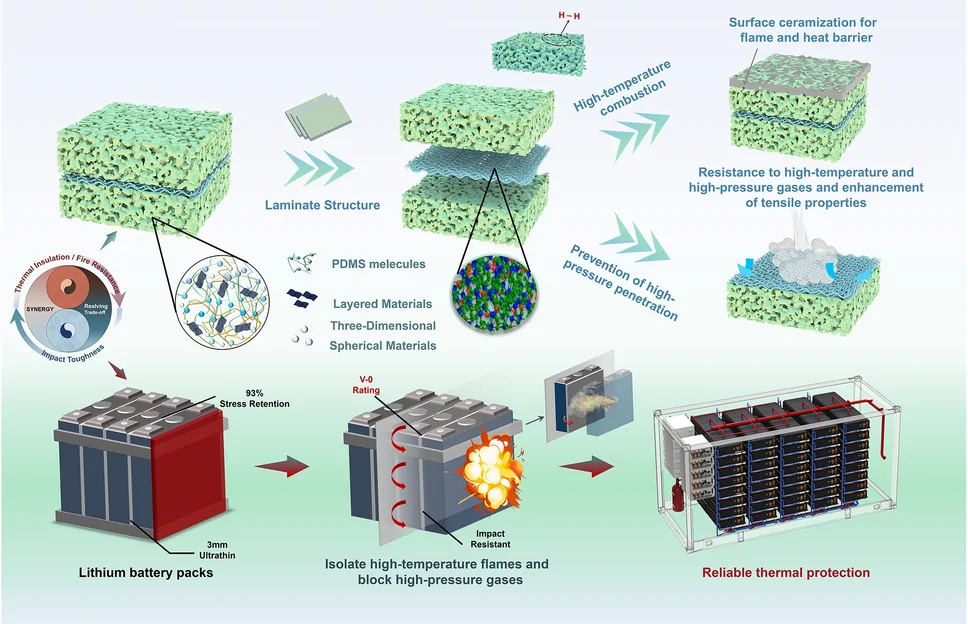

## Introduction

Driven by global carbon neutrality initiatives and the rapid transformation of energy infrastructures, lithium-ion batteries (LIBs) have become a pivotal energy storage solution [1, 2]. Their high energy density, long cycle life, and versatile integration capabilities position them at the forefront of renewable energy storage and electric vehicle proliferation [3]. However, as battery systems advance toward higher energy densities, safety incidents triggered by thermal runaway have escalated markedly [4–7]. Thermal runaway in LIBs is a complex, multiphysics-coupled dynamic process, typically initiated by abuse conditions such as overcharging, internal short circuits, or heat dissipation failure. This cascade generally evolves through three phases: thermal initiation, side reaction amplification, and abrupt energy release [8]. The final stage generates high-velocity, ultrahigh-temperature gas jets with core temperatures reaching 800–1200 °C, ejection velocities exceeding 200 m s^−1^, and a heat flux surpassing 50 kW m^−2^. These high-enthalpy jets directly elevate the temperature of neighboring cells via convective and radiative heat transfer [9–11], causing surface temperatures to spike by 300–500 °C within 0.1 s—far exceeding safe operating thresholds. Simultaneously, the dynamic pressure and shockwaves induced by high-velocity flow deform cell enclosures and dislodge electrode laminates, structurally compromising adjacent cells and lowering their thermal runaway thresholds. Moreover, the ejected combustible species mix with ambient air (or oxygenated byproducts) to form explosive mixtures, triggering secondary combustion at elevated temperatures [12–14]. This exothermic feedback loops accelerates Thermal Runaway Propagation (TRP) across the pack [15]. Experimental evidence indicates that gaseous jets from a single failing cell can rapidly induce sequential failure in multiple adjacent cells within seconds, establishing a self-propagating cascading failure that ultimately destroys the entire module [16]. Consequently, unraveling the mechanisms of TRP and developing advanced thermal protective materials are pivotal challenges in ensuring the safety of next-generation energy storage systems.

To mitigate battery thermal runaway, current thermal management strategies predominantly rely on a combination of passive thermal insulation and active heat dissipation [17]. Furthermore, integrating advanced sensor technologies for real-time monitoring and early warning has emerged as a crucial complementary strategy to proactively prevent catastrophic battery failures [18]. Additionally, advanced thermal regulation systems utilizing phase transition materials have recently garnered significant attention due to their exceptional latent heat storage capabilities and dynamic temperature control mechanisms [19–21]. As the primary defensive barrier, the efficacy of propagation suppression hinges critically on the properties of the passive insulation. Nevertheless, conventional materials struggle to maintain integrity under the extreme thermal and mechanical stresses imposed by high-temperature, high-pressure gas jets. Despite their low thermal conductivity (0.02–0.04 W m^−1^ K^−1^) and cost benefits, organic foams like polyurethane (PU) [22] and polystyrene (PS) encounter severe bottlenecks in battery safety applications [23]. Their limited thermal stability leads to rapid degradation above 300 °C, characterized by molecular chain scission and the release of combustible volatiles. Upon exposure to extreme heat (> 400 °C), these foams suffer from catastrophic structural integrity loss and decomposition [24]. The resulting residues (or voids) exhibit drastically increased gas permeability, thereby failing to act as an effective barrier against the high-pressure, high-temperature jets typical of thermal runaway [25–27]. More critically, the liquefied residues from organic foams can obstruct heat dissipation channels, thereby exacerbating thermal accumulation. Although modified mineral wool and comparable inorganic materials offer superior fire resistance, their inherently brittle fibrous architecture renders them vulnerable to erosion under high-velocity gas impact. Inorganic fibrous layers, often lacking robust interfacial cohesion, are susceptible to severe mechanical erosion and structural disintegration under high-velocity gas impingement. The resulting mass loss creates physical voids that serve as direct conduits for gas jet propagation. Furthermore, these materials often exhibit poor thermal shock stability; rapid temperature fluctuations can trigger fiber fracture and structural disintegration, leading to a substantial degradation in thermal insulation performance. Recently, various advanced functional thermal protection materials, including nanostructured aerogel composites [28], conventional homogeneous silicone rubber foams [29], and emerging multi-layer structural insulators (such as mixed-dimensional assembled foam heterostructures and superelastic micro/nanofibrous sponges) [30–32], have been widely reported to mitigate TRP in battery modules [33]. While these materials exhibit excellent static thermal insulation, they face severe challenges under the extreme dynamic conditions of actual thermal runaway and long-term operation of energy storage systems. As highlighted in recent high-impact studies, highly porous aerogels typically lack the mechanical robustness required to withstand high-velocity gas jets and physical impact, rendering them susceptible to structural collapse [34, 35]. Furthermore, their inherent brittleness makes them prone to powder shedding (pulverization) and cracking under continuous mechanical vibrations during transportation and assembly. Similarly, pure homogeneous silicone foams struggle to maintain structural integrity and effectively prevent perforation when exposed to ultrahigh-temperature (> 800 °C) and high-pressure gas jets. Although multi-layer architectural designs have shown great potential in improving overall battery safety and thermo-mechanical stability, existing solutions still confront an intrinsic trade-off: materials excelling in high-temperature resistance typically lack dynamic impact toughness, whereas impact-resistant counterparts fail to maintain thermal stability. To overcome this universal static material dilemma, the designed composite employs a gradient-laminated architecture where the silicone matrix tightly impregnates the interstices of the glass fiber fabric (GFF) during the foaming process. This structural integration, combined with the inherently high tensile strength of the GFF, forms a robust secondary physical barrier that effectively resists high-pressure gas jet penetration, while the subsequent synergistic ceramization guarantees exceptional high-temperature thermal stability. To overcome this universal static material dilemma, the designed SF/GFF_APP-ZB-Aero-Kao_ composite employs a multi-layer architecture where the silicone matrix tightly impregnates the interstices of the GFF during the foaming process. This structural integration, combined with the inherently high tensile strength of the GFF, forms a robust secondary physical barrier that effectively resists high-pressure gas jet penetration, while the subsequent synergistic ceramization guarantees exceptional high-temperature thermal stability.

Polydimethylsiloxane (PDMS) foam features a hybrid semi-inorganic–organic architecture anchored by a siloxane backbone (–Si–O–Si–). This distinct molecular configuration confers exceptional flexibility and inherent heat resistance, positioning it as a key material for thermal insulation and sealant applications. Nevertheless, practical deployment is hindered by the foam’s tendency toward structural collapse and embrittlement under thermal shock, driven by the rapid degradation of organic pendant groups. Current optimization strategies primarily focus on diverse approaches, ranging from matrix reinforcement via high-content inorganic fillers (e.g., metal hydroxides) to surface shielding using 2D nanomaterials like graphene oxide (GO) or MXene, alongside intrinsic backbone modifications—such as introducing phenyl groups or boron elements—to enhance char formation and thermal stability [36–40]. Recently, significant breakthroughs have been made in homogeneous ceramifiable silicone foams. For instance, an exceptional pottery-inspired flexible silicone foam was recently reported, demonstrating outstanding long-term thermal protection under static fire conditions. However, in real-world battery TRP scenarios, thermal barriers must endure not only extreme heat but also violent, high-velocity gas jet. Pure homogeneous foams, despite their excellent static fire resistance, often lack the macroscopic tensile strength to resist dynamic perforation by such high-pressure jets. Therefore, integrating a load-bearing skeleton into a gradient-laminated architecture remains fundamentally necessary to bridge the gap between static fire-shielding and dynamic TRP impact resistance.

GFF is a high-performance inorganic textile, demonstrates exceptional resilience in high-pressure gaseous environments due to its superior thermos-mechanical properties. Its interwoven architecture forms a networked load-bearing system that efficiently dissipates impact energy through inter-fiber friction and stress distribution, thereby preventing localized structural failure. Leveraging the high elastic modulus of the constituent glass fibers (70–85 GPa), the fabric maintains minimal deformation (< 1% strain) under elevated pressure, preserving its integrity as a robust physical barrier against high-velocity gas jets [41]. Moreover, precise weaving control enables tailored porosity management of the substrate. Subsequent impermeable coating treatments effectively seal these interstitial voids, reducing gas permeability to negligible levels. This treated architecture provides superior hermetic sealing efficacy compared to conventional porous separators, ensuring a robust barrier against gas permeation. GFF also demonstrates exceptional conformability, facilitating fabrication into complex shapes via lamination or hybridization with functional matrices (e.g., silicone and aerogels) [42]. This adaptability allows for the construction of multi-layer barriers that simultaneously satisfy sealing and mechanical buffering demands. Economically, GFF presents a decisive advantage, offering a 40%-50% lower cost compared to advanced counterparts like aramid fabrics, which, combined with established manufacturing scalability, positions it as an ideal solution for mass-produced energy storage systems.

The extreme thermal and mechanical stresses imposed by thermal runaway gas jets constitute a major barrier to energy storage safety. Existing materials, often constrained by trade-offs between thermal and mechanical performance, fail to mitigate these multidimensional risks. To address this, we developed a gradient-laminated ceramifiable composite integrating a PDMS foam matrix with a GFF interlayer. Compared to traditional commercial aerogels, this architecture leverages a robust ceramification mechanism to deliver a unique combination of high-temperature resilience, exceptional dynamic impact toughness, and mechanical durability (anti-pulverization), effectively withstanding the coupled ‘high-temperature–high-pressure’ environment. Consequently, this material is highly preferred for practical large-scale energy storage scenarios, where enduring assembly vibrations and resisting violent TR gas jets are critically demanded. As a scalable and effective protective strategy, this technology is poised to play a vital role in next-generation energy storage, supporting the global transition toward carbon neutrality.

## Experimental Section

### Materials

Materials and reagents were procured as follows: Divinyl-terminated polydimethylsiloxane (PDMS-Vi, viscosity: 10,000 cSt), hydroxyl-terminated polydimethylsiloxane (PDMS-OH, viscosity: 5,000 cSt), hydrogen-containing polydimethylsiloxane (PDMS-H, hydrogen content: 2.1%), and Karstedt’s platinum catalyst (5,000 ppm) were purchased from Hubei Shuanyauli Gao Biomedical Co., Ltd. Inhibitor (2,4,6,8-tetramethyl-2,4,6,8-tetravinyl-1,3,5,7,2,4,6,8-tetraoxatetrasilioxane) was obtained from Beijing Xinbaohai Chemical Technology Co., Ltd., which prevents immediate crosslinking network formation to allow sufficient mixing time for homogeneous distribution of prepolymers and components. Kaoline (Kao), Ammonium Polyphosphate (APP), and Zinc Borate (ZB) were sourced from Shanghai Aladdin Biochemical Technology Co., Ltd. Silica aerogel (Aero) was supplied by Tuwang New Material Co., Ltd., while silane coupling agent KH550 was acquired from Kangjin New Material Co., Ltd.

### Preparation of SF Foam, GFF and SF/GFFAPP_-ZB-Aero-Kao_

#### Fabrication Process of SF Foam

The preparation procedure was conducted as follows: Initially, 8 g of PDMS-H, 40 g of PDMS-OH, and the inhibitor were loaded into a reaction vessel and mechanically mixed at 900 rpm for 3 min until homogenization. Subsequently, 40 g of PDMS-Vi was introduced into the mixture, and the stirring rate was increased to 1,200 rpm for an additional 5 min to obtain a uniform prepolymer solution. Thereafter, 0.4 g of Karstedt’s catalyst (dosed at 5,000 ppm by mass) was injected into the prepolymer system, followed by rapid stirring for 30 s to activate the catalytic process. The activated formulation was then transferred into predesigned molds and allowed to stand at room temperature for 15 min to develop a three-dimensional network structure via foaming. Finally, the foamed specimens were subjected to heat treatment at 60 °C for 4 h in a constant–temperature oven to facilitate complete crosslinking and curing of the organosilicon network.

#### Modification Process of GFF

First, GFF sheets were cut into appropriate dimensions and dried in an oven at 80 °C for 2 h to eliminate moisture content. After removal from the oven, the surfaces were coated with KH550 silane coupling agent and subsequently allowed to stand undisturbed for 20 min, yielding the modified GFF composite.

#### Fabrication Procedure for SF/GFFAPP_-ZB-Aero-Kao_

First, 3 g of PDMS-H, 15 g of PDMS-OH, and the inhibitor were mechanically mixed at 900 rpm for 3 min to achieve homogenization. Subsequently, 15 g of PDMS-Vi, 0.5 g of APP, ZB, 5 g of Kao, and 0.1 g of Aero gel were incorporated into the solution, followed by vigorous stirring at 1,200 rpm for 5 min to obtain a uniform mixture. Thereafter, 0.15 g of Karstedt’s catalyst (dosed at 5,000 ppm by mass) was added, with rapid stirring for 30 s to activate the curing process. The resulting formulation was transferred into molds, where modified GFF sheets were pressed into the prepolymer matrix and allowed to foam at room temperature for 15 min. The composite material was then thermally cured in an oven at 60 °C for 2 h. Identical processing conditions were applied to fabricate the counterpart side of the SF/GFF_APP-ZB-Aero-Kao_ composite. Analogous preparation protocols were employed for the SF/GFF_APP-ZB_, SF/GFF_APP-ZB-Aero_, and SF/GFF_APP-ZB-Kao_ series, which shared identical compositional frameworks with SF/GFF_APP-ZB-Aero-Kao_.

### Material Characterization Methods

Microstructural analysis of the ceramic foam and other samples was conducted using scanning electron microscopy (SEM) via Zeiss UltraPlus and HITACHI S-4800 instruments. Full survey scans with high-resolution elemental profiling were performed via X-ray photoelectron spectroscopy (XPS) on a Thermo Scientific ESCALAB 250Xi system. X-ray diffraction (XRD) patterns were recorded on a Rigaku D/Max 2550 V diffractometer (Japan) over 10–70° 2θ range at 5° min^−1^ scanning rate. Thermal stability was evaluated through thermogravimetric analysis (TGA) using a Q500 analyzer. Mechanical properties including cyclic compression and tension were tested on a ZQ-950LB universal testing machine, with compression tests conducted on 20 × 20 × 20 mm^3^ specimens at 0.5 mm min^−1^ crosshead speed. Cone calorimetry tests complying with ASTM E1354/ISO 5660 standards were performed using an FTT UK device under 50 kW m⁻^2^ heat flux, employing 100 × 100 × 10 mm^3^ samples. Infrared thermography was carried out using a FOTRIC camera (measurement range: − 20–1500 °C) with emissivity set to 0.98. Ablation resistance was assessed in a custom-designed apparatus using 100 × 100 × 20 mm^3^ foam samples, with butane flame temperature and pool surface temperature monitored by K-type thermocouples (range: 0–1,200 °C). An HC-074–200 instrument operating on the steady-state method was employed to quantify the thermal conductivity of the SF/GFF_APP-ZB-Aero-Kao_ composite. To further evaluate the combustion behavior, limiting oxygen index (LOI) values were measured using a JF-3 device in compliance with ISO4589-2:1996 (specimen dimensions: 100 × 10 × 10 mm^3^). Finally, vertical flammability was assessed on a 5402H-V BURNING TESTER following the ASTM D 3801 protocol, utilizing samples sized 130 × 13 × 10 mm^3^.

### Battery TRP Testing Protocol

This study conducted systematic testing on the thermal runaway propagation characteristics of lithium-ion battery modules, and specifically investigated a commercial prismatic battery with a nominal capacity of 37 Ah. The battery employed LiNi_0.5_Co_0.2_Mn_0.3_O_2_ as the cathode material and graphite as the anode material, with overall dimensions of 148 × 27 × 91 mm^3^. It contained two jelly rolls, and the charge and discharge voltage cut-offs were 4.2 and 2.75 V, respectively. Prior to experimentation, individual cells underwent standardized pretreatment procedures: following complete discharge at a constant current, they were immediately subjected to full charging via a constant current–constant voltage (CC-CV) protocol, followed by a 24-h rest period to establish chemical equilibrium. The experimental setup utilized a three-cell module configuration, with insulating layers interposed between units to mitigate short-circuit risks. The assembly was mechanically stabilized using steel fixtures, incorporating ceramic fiber mats at contact interfaces to suppress unwanted thermal conduction. A multimodal synchronous data acquisition strategy was implemented: thermocouples (denoted as T_n,f_/T_n,b_) were positioned at the geometric centers of each cell’s front and back surfaces for real-time temperature field monitoring; high-precision data loggers captured voltage–time profiles simultaneously; and the entire module was placed on an analytical balance enabling mass measurement at 1 Hz frequency. Thermal runaway initiation was defined by a temperature rise rate exceeding 5 °C s^−1^, with a 300 W heating plate serving as the thermal stimulus source—heat application ceased immediately upon triggering thermal runaway. The experimental process was documented continuously through a high-speed imaging system. Comprehensive safety performance evaluation was achieved through integrated analysis of mass loss curves, temperature evolution profiles, and voltage response characteristics.

## Results and Discussion

### Design of Multi-layer Composite Thermal Insulation Material Based on Laminated Architecture

**Fig. 1:**
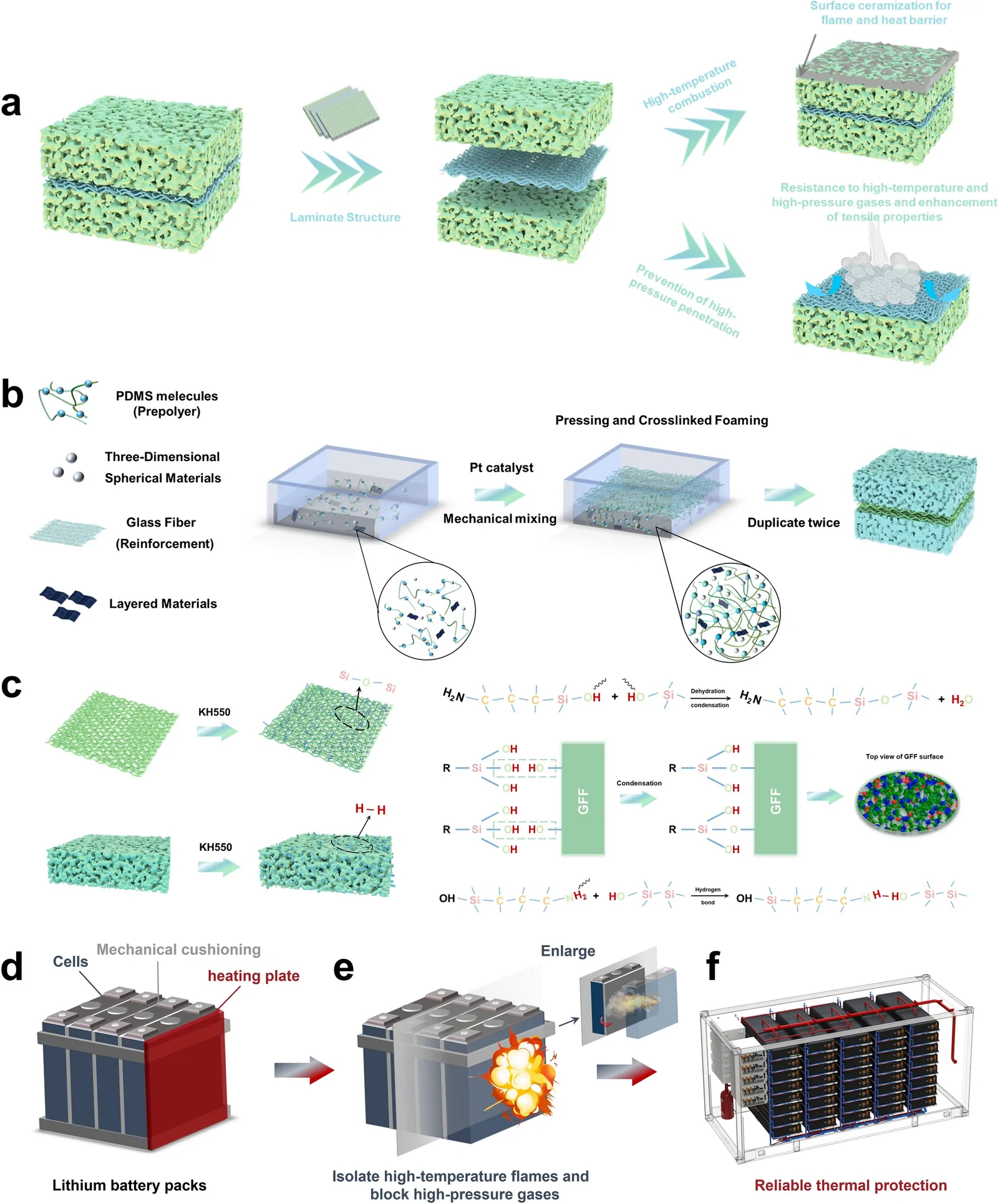
Design and fabrication of multiscale thermal insulation materials. **a** Schematic illustration of material functionality under high-temperature and high-pressure gas jetting conditions. **b** Flowchart for composite material preparation; **c** Schematic diagram of silane coupling agent pretreatment mechanism. **d-f** Potential application of SF/GFF_APP-ZB-Aero-Kao_ as a thermal insulation material in energy storage power stations

During the thermal runaway of LIBs, the violent ejection of high-temperature, high-pressure gas jets poses a severe threat, often perforating thermal insulation layers and triggering cascading failures in adjacent cells [4, 11, 16]. To address this critical challenge, PDMS foam has garnered significant research interest owing to its inherent thermal stability, electrical insulation, and exceptional mechanical flexibility. By synergizing the mechanical buffering capacity of laminated structures with the high-temperature resilience of ceramic materials, we propose a novel composite thermal insulation material (Fig. 1a). This design integrates a flexible PDMS matrix with functional fillers through a gradient-layered architecture. The resulting engineered material simultaneously achieves stable elasticity across a broad temperature range, robust fire resistance, and superior impact toughness. The primary objective is to overcome the vulnerability of conventional polymer foams to structural collapse and penetration under impinging gas jets, thereby fulfilling the critical demand for multifunctional materials capable of withstanding the extreme coupled thermal and mechanical shocks of thermal runaway.

Initially, ceramifiable silicone foam composites were fabricated via a reactive chemical foaming technique. The matrix, derived from a PDMS-based organic–inorganic hybrid precursor, possesses a Si–O–Si backbone ideal for ceramic conversion. The porous architecture was engineered through a dual-reaction mechanism catalyzed by Platinum (Pt), where gas evolution via the dehydrogenation condensation of Si–H (from PDMS-H) and Si–OH (from PDMS-OH) groups occurred concurrently with network curing via the hydrosilylation of Si–H and vinyl groups (from PDMS-Vi) (Fig. 1b). To reinforce this matrix, a silane-modified GFF interlayer was introduced. During foaming, the GFF was in situ integrated into the expanding polymer surface. The coupling agent played a pivotal role by establishing interfacial chemical bridging (Fig. 1c), which, combined with the mechanical interlocking facilitated by the fabric’s porosity, ensured exceptional adhesion between the GFF and the silicone matrix [43]. When the primary insulation is compromised by burn-through or high-pressure gas jetting, the GFF interlayer serves as a robust structural skeleton, effectively mitigating mechanical erosion and acting as a physical firewall. Complementing this physical reinforcement, the functional fillers activate a chemical defense mechanism. Zinc borate (ZB) and ammonium polyphosphate (APP) act as intumescent flame retardants, releasing inert gases during decomposition to dilute combustible volatiles and suppress surface combustion. Simultaneously, Kaolin (Kao) and silica aerogel (SiO₂ Aero) undergo high-temperature transformation; assisted by the fluxing effect of phosphate derivatives, they facilitate liquid-phase sintering to generate a dense ceramic barrier. This integrated multi-layer design prevents catastrophic failure caused by single-layer degradation, establishing a synergistic protection system. Consequently, the composite effectively shields adjacent cells from thermal shock, significantly mitigating the risk of propagation. This innovation offers a scalable and effective solution for thermal protection in energy storage power stations (Fig. 1d–f).

### Material Characterization and Structure Analysis

Based on the optimized processing system, an ultra-thin adhesive foam material was successfully fabricated. Leveraging its minimal thickness and excellent adhesive properties, this material achieves intimate and uniform interfacial bonding with battery surfaces during application, effectively eliminating interfacial defects caused by thickness heterogeneity. Simultaneously, its exceptional flexibility and processability enable precise tailoring into complex geometries according to specific application requirements, significantly expanding its practical utility across diverse scenarios.

**Fig. 2:**
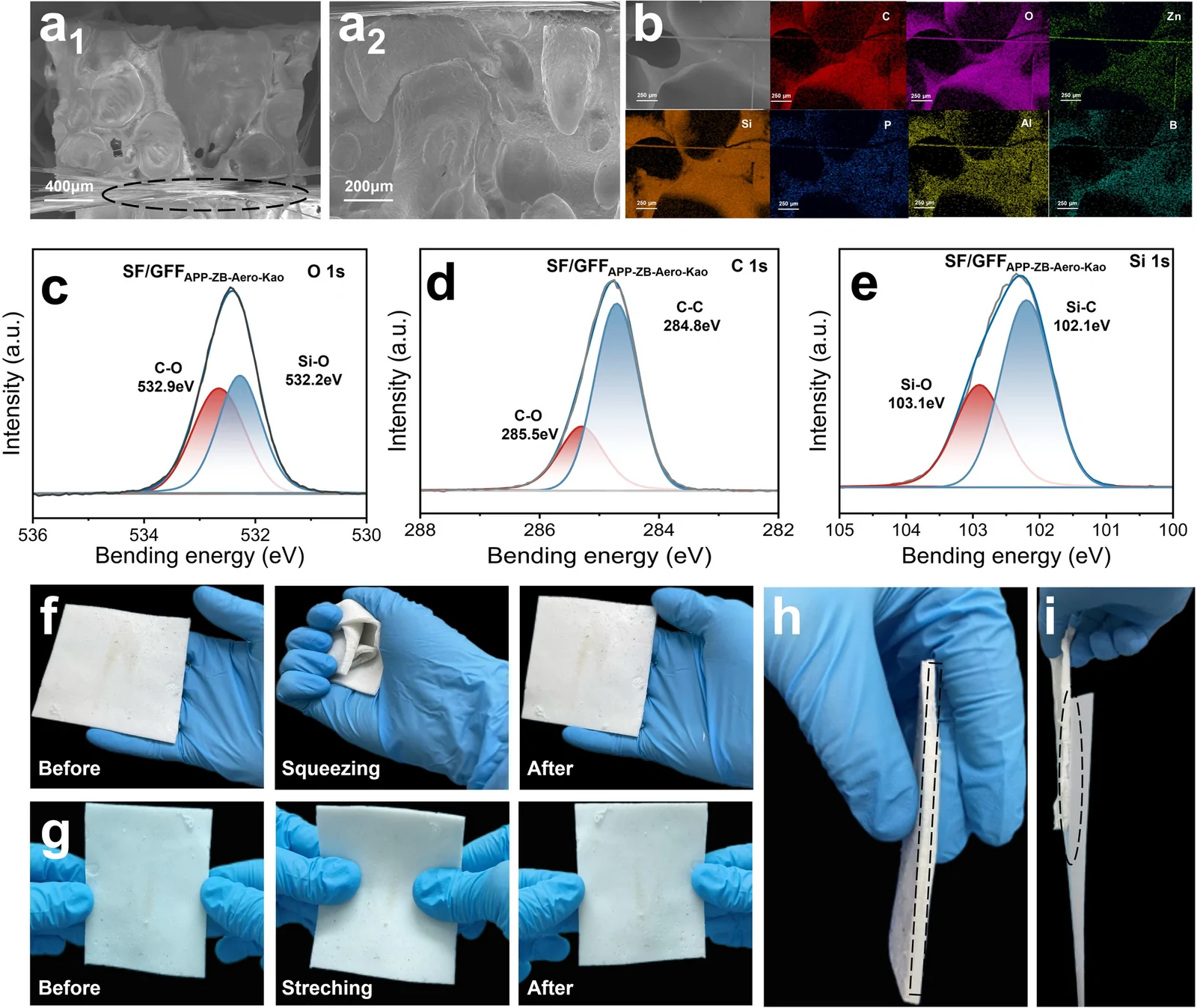
Microstructural morphology, chemical composition, and macroscopic physical properties. **a**_**1**_**, a**_**2**_ Cross-sectional SEM images of SF/GFF_APP-ZB-Aero-Kao_ at different magnifications. **b** EDS elemental mapping of selected regions in SEM.** c** O 1*s* XPS spectrum, **d** C 1*s* XPS spectrum, and **e** Si 2*p* XPS spectrum. **f, g** Optical images of SF/GFF_APP-ZB-Aero-Kao_ under various deformation modes and **h** optical image of fabricated 3 mm-thick panel and **i** optical image demonstrating excellent adhesive properties

To comprehensively investigate the structural compactness of the matrix material and interlayer bonding characteristics within the SF/GFF_APP-ZB-Aero-Kao_ laminated architecture, this study employed scanning electron microscopy (SEM) for high-resolution imaging analysis. The SEM observations revealed (Figs. 2a1, a2 and S1c-e) that the foam exhibits a typical closed-cell microstructure with cell diameters ranging from 35 to 100 μm. Notably, favorable graft interactions were observed between the internal fillers and the matrix framework, directly contributing to the formation of an irregular and rough surface morphology. Such microstructural features not only significantly enhance interfacial adhesion within the material but also establish a critical structural foundation for its superior mechanical performance. Regarding interlayer bonding properties, SEM images distinctly demonstrated intimate interlayer integration without observable voids or delamination. This exceptional bonding state originates from two synergistic mechanisms: (i) Silane coupling agent treatment facilitated the formation of Si–O–Si bonds and hydrogen bonding, substantially improving interlayer adhesion; (ii) During foam expansion, GFF were uniformly pressed into the material surface, creating embedded integration between cells and GFF, while PDMS progressively filled the interstices of the GFF, collectively reinforcing the overall structural stability. Energy-dispersive X-ray spectroscopy (EDS) was subsequently employed to perform elemental mapping and total spectrum analysis on the specimens. The test results demonstrated (Figs. 2b and S1f) that elements C, Si, and O were uniformly distributed throughout the foam matrix, confirming its organic siloxane backbone composition. The presence of Si and O within cellular pores validated the successful incorporation of SiO_2_ aerogel fillers. Notably, functional elements including Al, Zn, B, and P exhibited synergistic co-distribution patterns along pore walls. This phenomenon indicates that during material fabrication, these elements formed stable, homogeneous enrichment layers via chemical bonding or physical adsorption mechanisms, providing a critical chemical foundation for enhanced material performance. X-ray photoelectron spectroscopy (XPS) was employed to characterize the crosslinking state of the material. To elucidate elemental chemical environments, high-resolution XPS spectra of O, C, and Si were analyzed (Fig. 2c-e). Peak fitting revealed distinct chemical environments: in the O 1*s* spectrum, C–O bonds (532.9 eV) and Si–O bonds (532.2 eV) displayed characteristic peaks; in the C 1*s* and Si 2*p* spectra, C–C bonds (284.8 eV) and C–Si bonds (102.1 eV) manifested as discrete peaks [36]. These findings, corroborated by high-magnification imaging, confirmed successful crosslinking of the external thermal insulation layer, forming a stable three-dimensional network structure. Furthermore, to experimentally validate the chemical interaction mechanisms proposed during the foaming process (as illustrated in Fig. 1b), Fourier-transform infrared spectroscopy (FT–IR) was employed to track the structural evolution from the individual prepolymers to the cured matrix. As shown in Fig. S2a, the characteristic absorption peaks of the Si–H stretching vibration (at ~ 2160 cm⁻^1^) in H-PDMS, the vinyl –CH = CH_2_ stretching vibration (at ~ 1600 cm⁻^1^) in Vi-PDMS, and the broad –OH stretching band (at ~ 3460 cm⁻^1^) in OH-PDMS were distinctly observed in the respective prepolymers. However, in the spectrum of the cured silicone foam (SF), these characteristic reactive peaks almost completely disappeared, while the intensity of the Si–O–Si stretching vibration (at ~ 1020–1100 cm⁻^1^) significantly broadened and strengthened. This molecular-level evidence directly confirms the successful occurrence of both the hydrosilylation and dehydrogenation condensation reactions. Additionally, to experimentally verify the interfacial chemical bridging mechanisms during the GFF modification process (as proposed in Fig. 1c), both FT–IR and XPS analyses were conducted. In the newly obtained FT–IR spectra (Fig. S2b), compared with the pristine GFF, the KH550-modified GFF exhibited distinct new characteristic peaks at 2925 and 1560 cm⁻^1^, which correspond to the alkyl chains (–CH_2_–) and amino groups (–NH2) of the silane coupling agent, respectively. This surface modification was further corroborated by the high-resolution XPS N 1* s* spectrum (Fig. S2c), which revealed a characteristic N–H binding energy peak at 399.7 eV. These combined molecular-level results strictly confirm the successful grafting of KH550 onto the GFF skeleton. Mechanical property testing further revealed exceptional mechanical flexibility and tensile strength, with the material demonstrating remarkable resistance to plastic deformation under intense mechanical loading (Figs. 2f, g and S1a, b), thereby ensuring durability and reliability in practical applications. Significantly, the laminated SF/GFF_APP-ZB-Aero-Kao_ architecture enables fabrication of ultra-thin 3 mm-thick panels (Fig. 2h) with superior adhesive properties, ensuring intimate conformal contact with battery modules during service (Fig. 2i).

### Characterization of Mechanical and Thermal Insulation Performance of the Material

Prior to evaluating the mechanical and thermal insulation behaviors, the bulk densities of the materials were measured. Interestingly, the pristine SF exhibits a bulk density of 333 mg cm^−3^, whereas the optimized SF/GFF_APP-ZB-Aero-Kao_ composite shows a noticeably decreased density of 309 mg cm^−3^. This highly beneficial density reduction is mainly ascribed to the incorporation of the ultra-lightweight aerogel, which significantly dilutes the mass of the solid matrix. Furthermore, the embedding of the GFF skeleton and multiscale fillers introduces additional interfacial micro-voids and free volume within the structure, collectively lowering the overall bulk density. Consequently, this remarkably lightweight profile perfectly fulfills the stringent mass-saving requirements for practical energy storage systems.

**Fig. 3:**
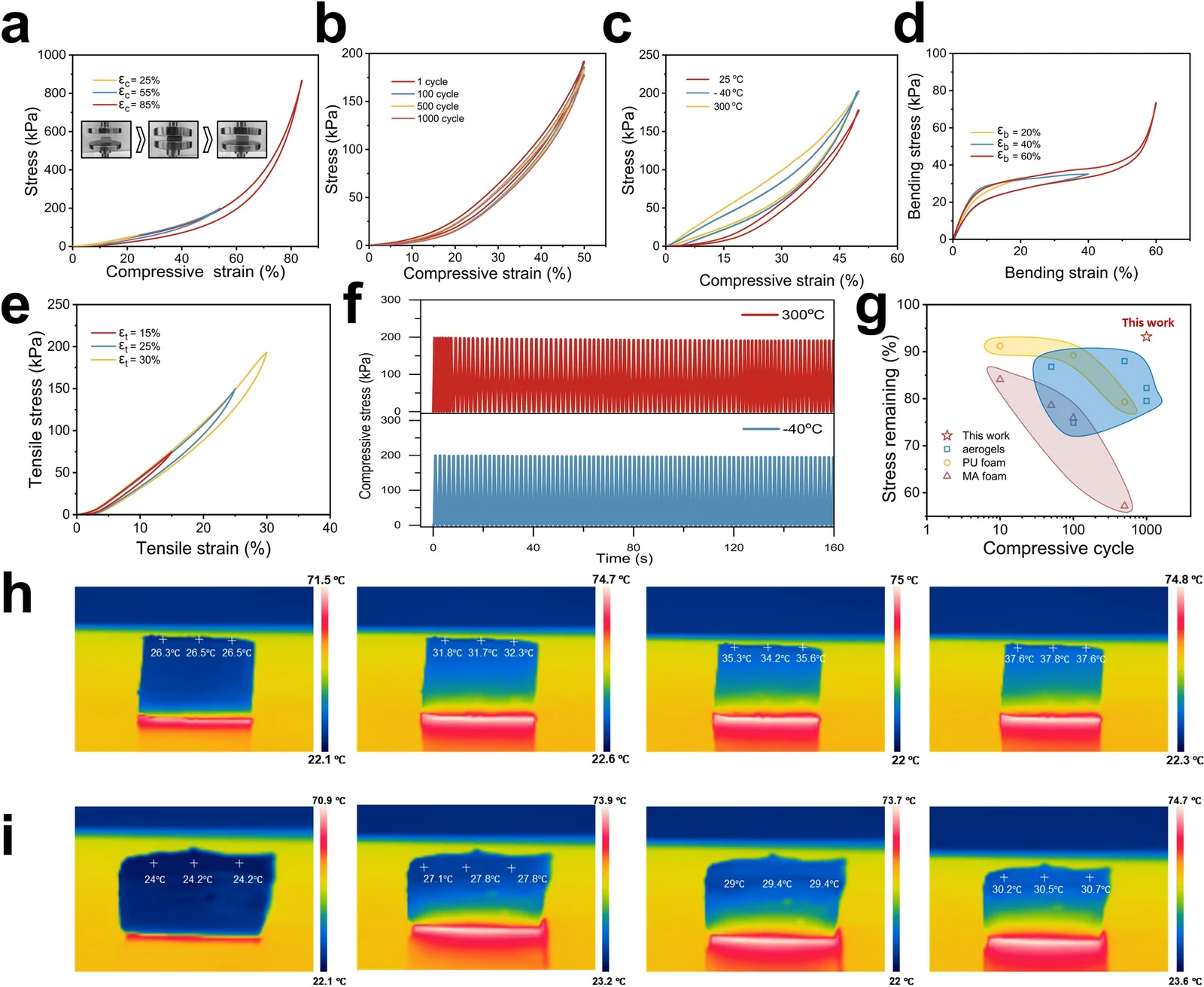
Mechanical robustness, extreme-temperature fatigue resistance, and thermal insulation performance. **a** Uniaxial compression up to 85% strain; inset: snapshot of one loading cycle. **b** Compressive stress–strain curves for 1000 cycles at 50% strain; **c** Compressive stress–strain curves at – 40, 25, and 300 °C under 50% strain. **d** Bending test with strain up to 60%. **e** Tensile test with strain reaching 30%. **f** Evolution of compressive stress during continuous cyclic compression at 50% strain for 160 min at 300 °C and − 40 °C. **g** Comparison of residual stress under cyclic compression with other reported elastic porous materials. **h, i** Infrared thermal images of SF/GFF_APP-ZB-Aero-Kao_ during sustained heating at 80 °C

The study conducted an in-depth investigation into the material’s compressive elasticity and fatigue resistance under both ambient and extreme operating conditions, alongside comprehensive evaluations of its flexural flexibility and tensile properties [44]. Regarding compressive resilience, analysis of stress–strain curves within a 25%–85% deformation range (Fig. 3a) revealed exceptional recovery behavior of the SF/GFF_APP-ZB-Aero-Kao_ composite. Even at 85% compressive strain, complete shape restoration occurred post-load release, accompanied by a peak stress of 866 kPa. For fatigue characterization, cyclic compression testing (1,000 cycles at 50% strain, room temperature) demonstrated significantly reduced stress degradation compared to pristine SF materials, maintaining ~ 93% residual stress after cycling (Fig. 3b). Notably, stress diminution primarily occurred during the initial 1–100 cycles, with < 5% residual strain reduction between the 100th and 1000th cycle, substantiating superior fatigue endurance. To assess extreme environment performance, tests were performed across a broad temperature spectrum (− 40 to 300 °C). As shown in Fig. 3c, thermal extremes exerted minimal influence on material stress responses. At cryogenic temperatures, marginal stress elevation was attributed to augmented internal friction mechanisms. Subsequent long-duration cyclic compression (50% strain, 160 min) at both 300 and –40 °C (Fig. 3f) yielded fatigue resistance and resilience metrics comparable to ambient conditions, indicating negligible structural degradation under thermal stressors. Over 1,000 compression cycles, the material’s residual stress surpassed benchmark values for previously reported aerogels (blue region) [45–48], PU foams (yellow region) [49–51], and melamine (MA) foams (red region) (Fig. 3g) [52, 53], demonstrating exceptional elastic recovery. Furthermore, outstanding flexural flexibility and tensile capabilities were observed: maximum bending strain reached 60% (Fig. 3d) with corresponding stress of 78.4 kPa, signifying robust structural stability under flexural loading. Tensile testing revealed near 200 kPa stress at 30% strain without failure (Fig. 3e), enabled by glass fiber reinforcement, with full recovery upon stress release—outperforming SF foam counterparts in overall mechanical performance.

For thermal protective materials, besides mechanical properties, thermal insulation performance stands as another critical determinant of practical application value. To systematically evaluate the composite’s insulating capabilities, this study employed a thermal stability testing system: A constant temperature heating platform established an 80 °C steady heat source environment, while infrared thermography enabled real-time dynamic monitoring of surface temperature evolution. Comparative experimental results (Fig. 3h, i) revealed that pure SF foam exhibited substantial thermal conduction during initial exposure (0–10 min), with surface temperatures rapidly escalating from 26.5 to 32.3 °C — a temperature rise of 5.8 °C. After sustained heating for 30 min, the rear-side temperature reached 37.8 °C, yielding a total temperature increase of 11.3 °C. In contrast, the engineered SF/GFF_APP-ZB-Aero-Kao_ composite demonstrated exceptional thermal insulation under identical 30-min heating conditions, registering a mere 5.7 °C temperature variation—representing a ~ 50% reduction (5.6 °C decrease) in temperature rise compared to pure SF foam. This pronounced thermal differential conclusively validates the composite’s stable insulating performance (Supporting Information Table S1), which is fundamentally supported by its intrinsically low thermal conductivity (reduced from 0.094 W m^−1^ K^−1^ for pure SF to 0.046 W m^−1^ K^−1^ for the SF/GFF_APP-ZB-Aero-Kao_). Despite incorporating the GFF as a reinforcing interlayer, this significant decrease in overall thermal conductivity is primarily driven by the ultra-low intrinsic thermal conductivity of the aerogel. Additionally, the massive heterogeneous interfaces introduce extra thermal boundary resistance, while the sandwich-like layered architecture and interfacial micro-voids create a highly tortuous heat flow path and trap stagnant air. Collectively, these insulating factors completely outweigh the inherent thermal conductivity of the glass fiber interlayer. Consequently, the material maintained a steady temperature plateau throughout thermal exposure, indicative of its efficacy in impeding heat flux transmission and achieving superior thermal barrier functionality.

### Fire-Resistant Performance of Multiscale Synergistic Ceramicizing Materials

The cone calorimetry method, grounded in the oxygen consumption principle, establishes a controlled thermal irradiation environment to simulate real-world fire scenarios, enabling quantitative assessment of material flammability properties [54]. This technique plays a pivotal role in polymeric material flame retardancy research by replicating fire-induced thermal conditions and precisely measuring critical parameters during combustion, thereby providing essential data for in-depth analysis of material burning behavior. In this study, an innovative analytical approach was adopted by integrating ignition experiments with cone calorimetry to conduct a comprehensive investigation into the fire-resistant performance of diverse foam materials.

**Fig. 4:**
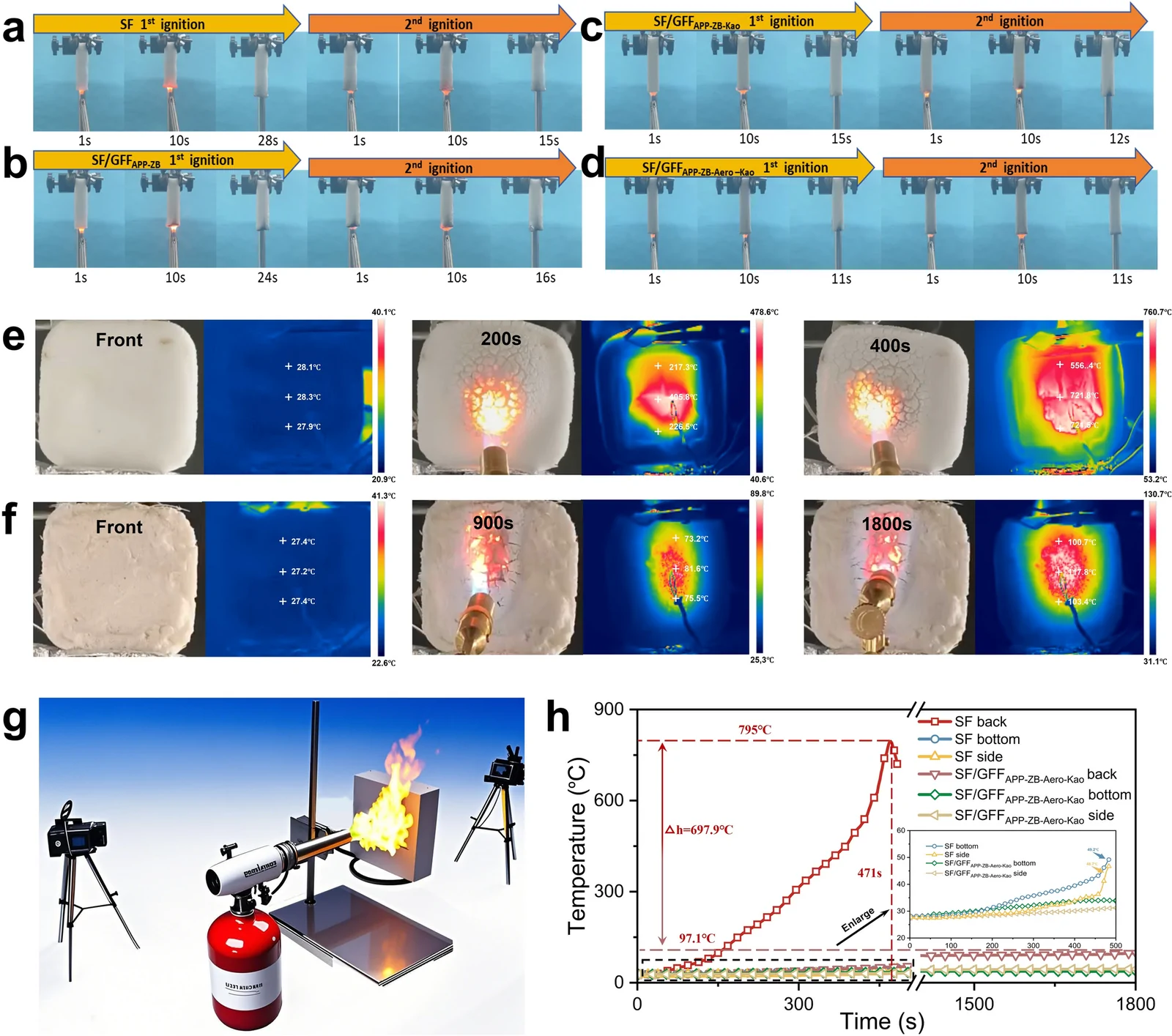
Flame retardancy, combustion behavior, and high-temperature fire resistance. **a** Ignition tests of SF, **b** SF/GFF_APP-ZB_, **c** SF/GFF_APP-ZB-Kao_, and **d** SF/GFF_APP-ZB-Aero-Kao_. **e** Optical images during combustion testing for SF and **f** SF/GFF_APP-ZB-Aero-Kao_, along with corresponding rear-side infrared thermographic images. **g** Schematic diagram of the custom-designed butane torch combustion testing apparatus. **h** Evolution curves of surface temperatures during combustion testing for SF and SF/GFF_APP-ZB-Aero-Kao_

The ignition test demonstrated that pure SF foam exhibited extremely poor fire resistance stability: It ignited immediately upon 2 s of flame exposure, sustained combustion for 28 s after external flame removal, accompanied by surface microcracking, edge region melting, and char spallation (Fig. 4a). In stark contrast, the composite system rapidly formed a dense ceramicized protective layer under high-temperature exposure, maintaining stable combustion interface morphology that fundamentally differed from the melting failure mode of pure SF. Incorporation of flame retardants extended the first ignition time to 8 s, with self-extinction occurring after 24 s of sustained burning (Fig. 4b). Post-incorporation of Aero gel powder, its exceptional high-temperature resistance combined with microscale protective layers formed during re-melting under flame-retardant action further reduced material reignition tendencies. With the addition of Kao laminar systems (Fig. 4c, d), multicomponent synergistic effects enabled self-extinction within 10 s of initial ignition and instantaneous self-extinction upon secondary ignition. Comparative examination of post-combustion specimen interfaces (Fig. S3a) revealed significant thermal erosion around SF foam samples, severe pyrolysis char layer delamination exposing porous structures and weak interfacial bonding—rendering them vulnerable to high-temperature flame impingement. Conversely, the SF/GFF_APP-ZB-Aero-Kao_ composite achieves a transformative shift from passive thermal insulation to active fire suppression. Indeed, consistent with the aforementioned ignition observations, standard flammability tests confirmed that the optimized composite achieved a high limiting oxygen index (LOI) of 33.5% and effortlessly passed the UL-94 V-0 rating, whereas the pristine SF merely exhibited an LOI of 25.5% and only attained a V-1 rating Fig. S4 and Table S2). This remarkable fire resistance is fundamentally driven by its multiscale synergistic flame retardancy (comprising nanoscale lamellar barriers, microscale ceramic frameworks, and macroscale char protective layers) coupled with a laminated structural design. Specifically, its protective functionality operates via dual mechanisms: firstly, a dense ceramic layer reduces heat transfer rates while isolating the material from direct flame impingement; secondly, nitrogen–phosphorus flame retardants suppress oxygen diffusion and curb the propagation of combustible gases. Together, these mechanisms significantly decelerate material thermodegradation and markedly enhance the overall fire resistance performance.

To enable visualizable and quantitative evaluation of the fire resistance performance of SF/GFF_APP-ZB-Aero-Kao_, this study established a specialized testing apparatus integrating an infrared thermal imager and multiple thermocouples (Fig. 4g). A high-definition digital camera documented the flame propagation dynamics, while real-time temperature monitoring was conducted across the foam block’s side interior, base, and rear surfaces using synchronized infrared thermography and strategically positioned thermocouples. The test employed a butane flame generating approximately 1100 °C—substantially exceeding the material’s initial melting point—to simulate extreme fire exposure conditions for comprehensive performance assessment.

During the initial phase of testing, pure SF exhibited extremely poor fire resistance stability. Upon brief flame exposure (within seconds), it underwent continuous expansion followed by intense combustion. At 200 s of burning, cracks emerged at the center region and progressively expanded, accompanied by a rear-side temperature escalation to 405.8 °C (Fig. 4e). Complete burn-through occurred after 400 s (Fig. S3b), with infrared thermography revealing localized temperatures exceeding 720 °C and thermocouples recording a peak rear-surface temperature of 795 °C. In stark contrast, the modified composite demonstrated exceptional fire resistance performance (Fig. 4f). Upon flame contact, its surface rapidly underwent ceramicization, forming a dense physical barrier that effectively obstructed heat transfer. After 15 min of ignition, the rear-side temperature increased only to 81.6 °C; even after 30 min of sustained flame impingement, the material maintained structural integrity (Fig. S3c) with no significant surface damage. The rear-surface temperature stabilized at 97.1 °C, while the internal side temperature rose to 45.1 °C (Fig. 4f, h), indicating low lateral thermal conductivity attributed to Aero filler incorporation. Minimal temperature variation was observed at the base. Apart from minor damage at the direct flame impingement zone, the material preserved its original macroscopic dimensions and structure (Fig. S3c). The remarkable enhancement in fire resistance primarily stems from the synergistic flame-retardant effects of APP and ZB under high-temperature conditions. During thermal decomposition, their active decomposition products interact with Kao through crosslinking reactions, promoting char layer formation and ultimately establishing a continuous, rigid ceramicized protective layer. This dense ceramic barrier significantly retards heat penetration into the material interior via efficient thermal blocking and mass transport suppression mechanisms. Crucially, when subjected to high-pressure flame jets generated by the burner, the embedded GFF provides effective resistance against flame penetration, further decelerating heat transfer rates and preventing material perforation.

**Fig. 5:**
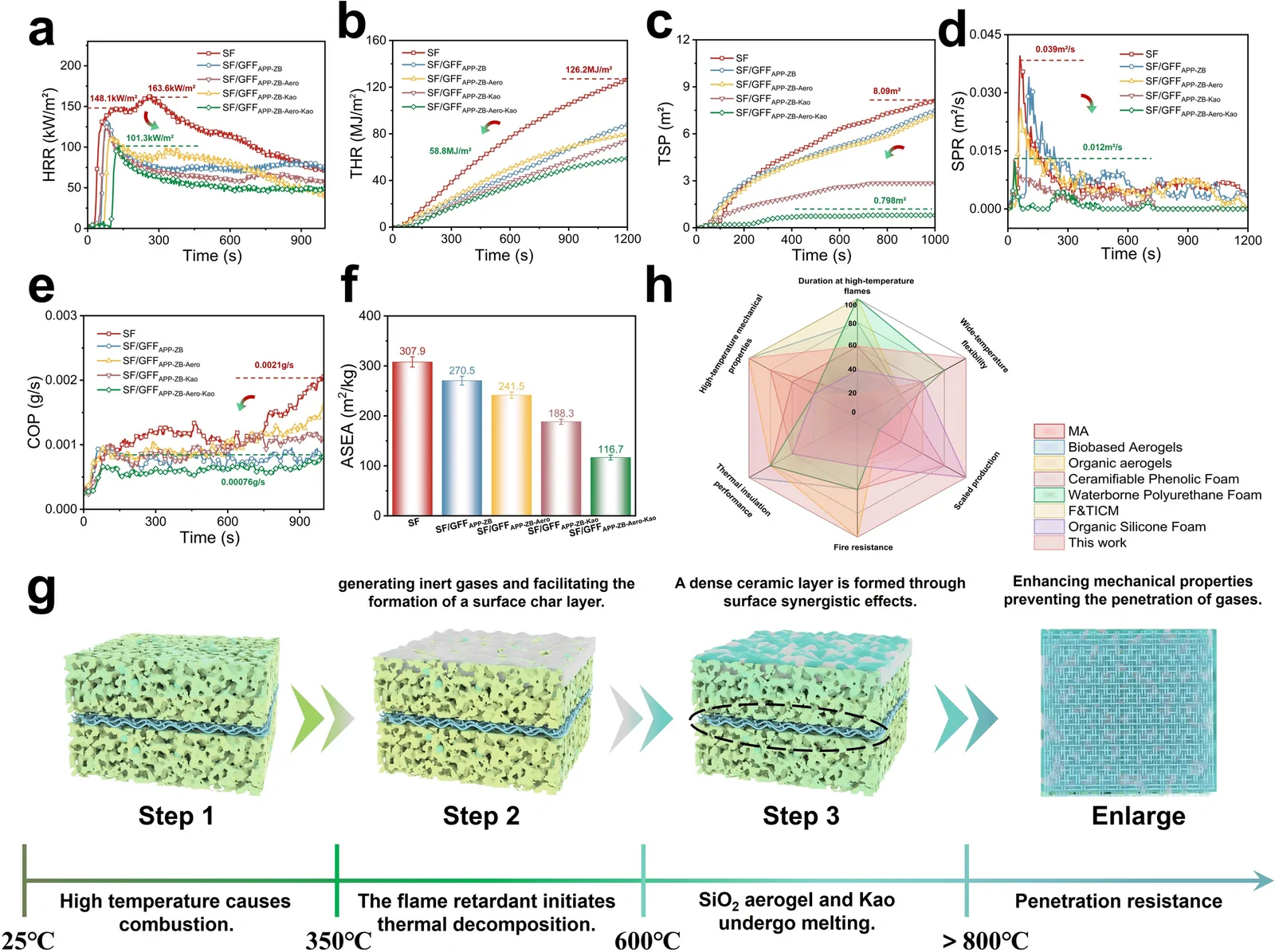
Fire hazard assessment and rapid synergistic ceramicization mechanism. **a** HRR, **b** THR, **c** TSP, **d** SPR, **e** COP, and **f** ASEA values from cone calorimetry tests; **g** Schematic illustration of the rapid ceramicization transformation mechanism under sustained high temperatures and internal resistance to jetted gases for SF/GFF_APP-ZB-Aero-Kao_; **h** Comparative performance of SF/GFF_APP-ZB-Aero-Kao_ against other high-temperature-resistant aerogels and foams

In the cone calorimetry tests, strict control of experimental conditions enabled precise measurement of critical combustion characteristics including Heat Release Rate (HRR), Smoke Production Rate (SPR), Total Smoke Production (TSP), Time to Ignition (TTI), and Average Specific Extinction Area (ASEA). These parameters collectively characterize material flammability, combustion behavior, and residue properties, revealing distinct burning patterns of different foam materials under fire scenarios. Regarding pure SF foam, its rapid ignition propensity was evident with a TTI of merely 27 s, accompanied by intense exothermic reactions during combustion. Experimental data confirmed its high flammability: The first peak HRR reached 148.1 kW m^−2^ at 122 s, followed by a secondary peak of 163.6 kW m^−2^ at 269 s (Fig. 5a), indicative of accelerated structural degradation. Consequently, the THR attained 126.2 MJ m^−2^ (Fig. 5b). In stark contrast, the flame-retardant-and-filler-modified system demonstrated significantly improved fire performance: TTI extended beyond 96 s, HRR reduced by 38.1% (peaking at 101.3 kW m^−2^), and near elimination of secondary combustion peaks, achieving a 54.4% reduction in THR. This enhancement is attributed to the dense ceramicized char layer formed on the burning surface, which physically retarded thermal decomposition—an observation corroborated by comparative analysis in Fig. S5a–e. Beyond thermal metrics, the modified system exhibited superior smoke suppression performance (Fig. 5c, d): TSP decreased to 0.798 m^2^ and SPR reduced to 0.012 m^2^ s^−1^, representing reductions of 87.9% and 69.2%, respectively, compared to pure SF foam. Regarding toxic gas emissions, experimental data demonstrated significant reductions in both CO and CO_2_ release rates for the modified material compared to pure SF (Figs. 5e and S3d). Peak values decreased from 2.1 mg s^−1^ (COP) and 67 mg s^−1^ (CO_2_P) for SF to 0.76 mg s^−1^ and 40.3 mg s^−1^ for the optimized system, representing reductions of 64% and 40.3%, respectively.

To quantitatively characterize combustion kinetics, Fire Performance Index (FPI) and Fire Growth Index (FGI) were introduced as evaluation metrics [54]. FPI, defined as the ratio of TTI to PHRR [FPI = TTI/PHRR], correlates negatively with intrinsic fire hazard; FGI, calculated as PHRR divided by time to peak heat release (tPHRR) [FGI = PHRR/tPHRR], reflects fire propagation rate. Results (Fig. S3e) showed that SF exhibited an FPI of 0.1403 m^2^ s kW^−1^ and an FGI of 1.477 kW m^−2^ s^−1^, whereas the composite system achieved a substantially improved FPI of 0.93 m^2^ s kW^−1^ and a reduced FGI of 0.78 kW m^−2^ s^−1^. This disparity demonstrates that the synergistic flame-retardant system effectively suppresses fire source risk coefficients and chain reaction rates, thereby significantly enhancing flame retardancy. Thermogravimetric (TG) analysis further corroborated these findings (Fig. S3f, g). Notably, the SF/GFF_APP-ZB-Aero-Kao_ composite demonstrated exceptional thermal stability with negligible weight loss up to 300 °C. Upon continuous heating to 1,000 °C, it revealed a significantly higher residual mass retention compared to pure SF, achieving a remarkably high ceramic residue yield of 64.97%. This quantified high solid residue directly substantiates the synergistic ceramification mechanism and the formation of a robust inorganic barrier under high-temperature conditions. More importantly, compared to conventional aerogels and composite porous thermal insulation materials, the SF/GFF_APP-ZB-Aero-Kao_ system exhibits comprehensive advantages including prolonged resistance under high-temperature flame exposure, superior high-temperature mechanical properties, enhanced fire resistance, and scalable manufacturing feasibility—positioning it as a promising candidate for practical thermal protection applications (Fig. 5h) [49, 55–60]. Based on experimental findings, Fig. 5g elucidates the material’s working mechanism and interlayer functionalities. Upon exposure to elevated temperatures, internal flame retardants undergo decomposition, releasing inert gases that dilute combustible vapors in the gas phase. Concurrently, these agents facilitate char layer formation on the material surface. As temperatures surpass 600 °C, Kao and Aero powders progressively dissolve and gradually integrate with the preformed char layer, generating a denser protective barrier against flame impingement. At temperatures exceeding 800 °C, complete dissolution of fillers occurs, enabling sustained crosslinking reactions with the char layer to ultimately arrest flame propagation. Notably, GFF plays a critical role: even when one side of the material is penetrated, GFF effectively blocks further flame invasion. Additionally, during exposure to high-velocity, high-pressure gas jets, GFF provides robust physical protection against perforation, thereby safeguarding underlying components from thermal hazards.

### Analysis of Ceramicization Mechanism

**Fig. 6:**
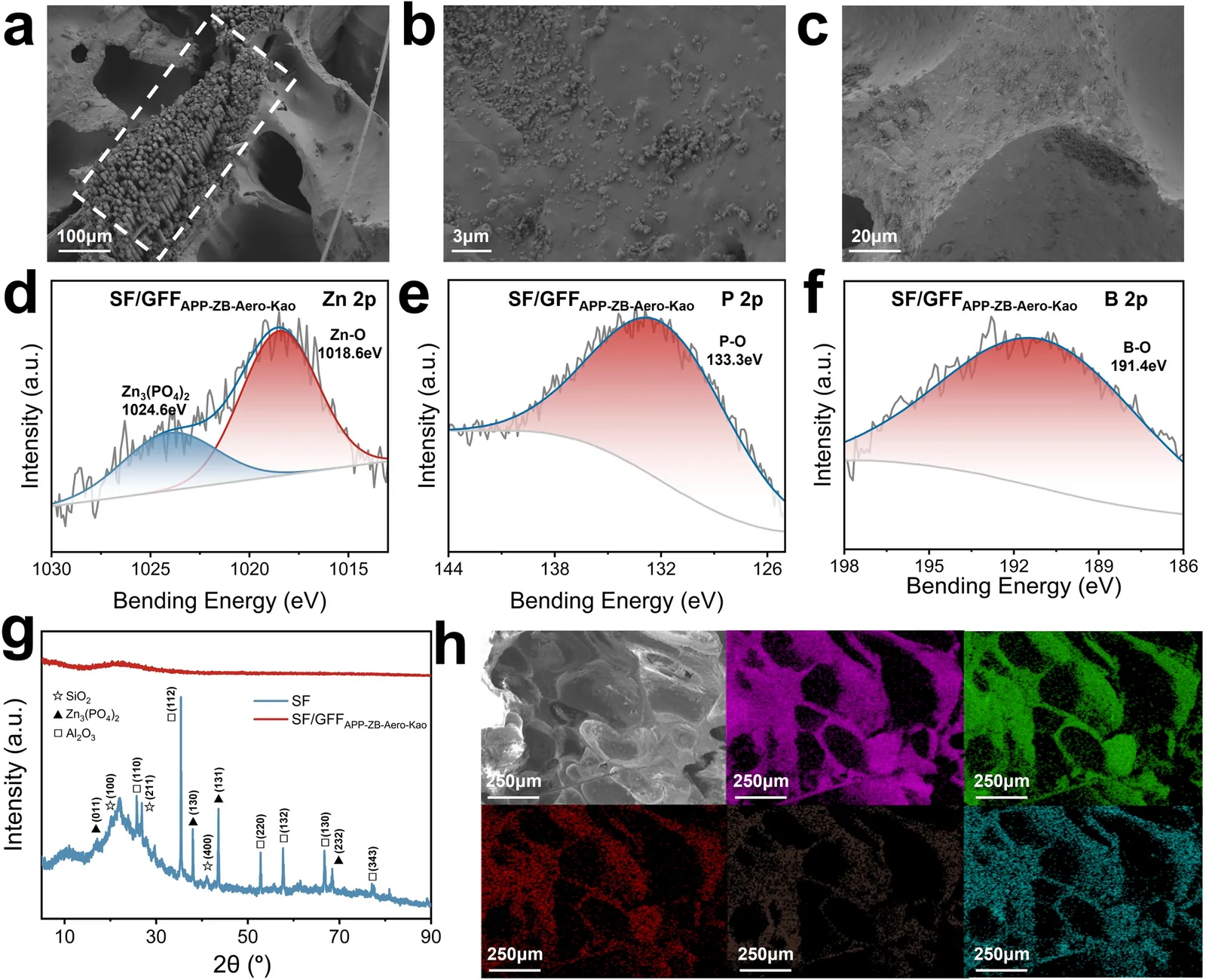
Microstructural, elemental, and phase evolution analysis of the ceramicized char residues. **a-c** SEM images of residual char from SF/GFF_APP-ZB-Aero-Kao_ after combustion testing; **h** Corresponding elemental mapping spectra of selected surface regions. **d** XPS Zn 2*p*, **e** P 2*p*, and **f** B 2*p* spectra of the char residue. **g** XRD patterns comparing SF and SF/GFF_APP-ZB-Aero-Kao_

To elucidate the mechanism underlying foam ceramicization at elevated temperatures, this study employed comprehensive characterization techniques including SEM, XRD, and XPS to systematically analyze post-cone-calorimetry surface char layers [27, 53, 61]. High-magnification SEM images (Fig. 6a-c) revealed pronounced densified sintering characteristics on the material surface, with the ceramicized sintered layer exhibiting significantly compacter morphology compared to pure SF systems. Notably, GFF remained structurally intact and embedded within the matrix post-testing, maintaining robust integration with the foam substrate. EDS elemental mapping (Fig. 6h) demonstrated continuous and uniform distribution of Si and C elements within the ceramicized products, corresponding to the siloxane backbone derived from PDMS pyrolysis and Aero gel decomposition residues. In contrast, Al, P, and Zn elements displayed discrete, homogeneous dispersion patterns, confirming active participation of intumescent flame retardants APP and ZB in high-temperature chemical reactions. Additionally, Kao underwent melting transition under thermal exposure, further promoting dense ceramic layer formation. Subsequent XPS analysis was conducted to investigate residual elemental chemical states on the post-combustion surface, thereby probing structural evolution mechanisms and the influence of APP/ZB on the ceramicization process.

The XPS analysis findings offer additional validation for the role of APP and ZB in driving the ceramicization process [62]. Spectral data revealed a characteristic peak at 126.2 eV in the P 2*p* orbital binding energy spectrum (Fig. 6e), corresponding to the P–O chemical state, thereby directly confirming APP’s participation in ceramicization reactions. Concurrently, distinct peaks observed in the Zn 2*p* orbital at 1024.6 and 1018.6 eV, along with a B 2*p* peak at 176.6 eV (Fig. 6d, f), substantiated ZB’s involvement in this transformation. Crucially, the binding energies of the P 2*p* orbital and the 1024.6 eV Zn 2*p* peak are assignable to the formation of an α-Zn_3_(PO_4_)_2_ glassy phase, suggesting potential melt synergies among APP, ZB, Kao, and Aero during high-temperature exposure. Analysis of XRD patterns (Fig. 6g) further corroborated these observations: while pristine SF exhibited no discernible diffraction peaks—indicative of its low crystallinity and predominantly amorphous structure—the ceramicized foam product displayed sharp characteristic peaks corresponding to Al_2_O_3_, SiO_2_, and Zn_3_(PO_4_)_2_, highlighting significant phase evolution. These results collectively demonstrate that during thermal exposure, polyphosphoric acid derived from APP decomposition reacts with ZB degradation products to form phosphate-containing glassy phases. Simultaneously, Aero integrates into the siloxane network via interfacial chemical interactions, while Kao transforms into SiO_2_ based components. This multiphase synergy establishes a composite ceramic layer anchored by a silicate framework, whereby Kao and Aero powders enhance high-temperature stability, and phosphate glassy phases promote structural densification. The resulting ceramicized barrier achieves synergistic optimization of thermal-oxidative resistance and mechanical reinforcement, facilitating the transition of surface char layers into ceramic phases under extreme temperatures.

### Prevention of Thermal Runaway Propagation

**Fig. 7:**
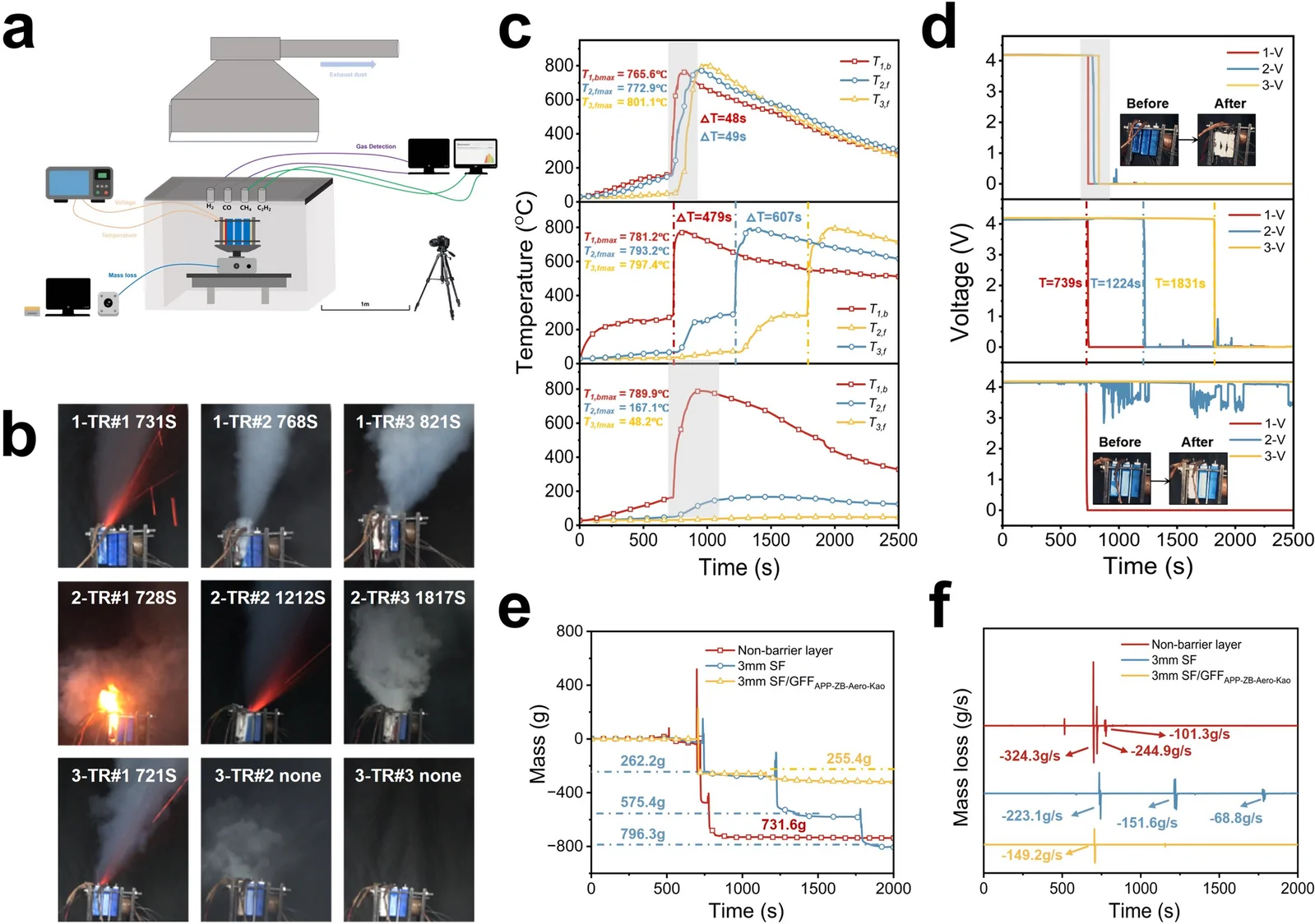
Evaluation of thermal runaway propagation (TRP) suppression performance in battery modules. **a** Custom-built TRP testing platform. **b** High-resolution videography capturing TRP progression for uninsulated, SF-insulated, and SF/GFF_APP-ZB-Aero-Kao_-protected configurations. **c** Temperature evolution curves on front/rear surfaces of battery cells across experimental groups. **d** Voltage fluctuation profiles of the battery module; Inset: High-resolution photographs of the module before/after testing. **e, f** Total mass loss and mass loss rate curves for the experiments

In practical engineering applications of lithium-ion battery modules, thermal runaway (TR) constitutes a critical safety concern demanding urgent resolution [61]. When individual cells undergo TR [63], rapid self-heating, pressure venting, and combustion occur within seconds, with most heat propagating to adjacent cells via thermo-mechanical-electrochemical coupling mechanisms until entire module failure—triggering cascading TRP and structural collapse. This hazardous process necessitates containment through engineered thermal barrier designs and protective architectures. To validate the protective efficacy of the SF/GFF_APP-ZB-Aero-Kao_ material, this study established a custom-built testing platform designed for practical battery module configurations (Fig. 7a), evaluating its performance in mitigating TRP based on empirical data. During experiments, high-definition cameras documented the entire TRP progression (Fig. 7b), while thermocouples and voltage sensors were strategically affixed to both front and rear surfaces of each cell for real-time monitoring of temperature fluctuations and voltage dynamics. Comparative analysis of module mass loss metrics alongside experimental datasets further corroborated the material’s protective capabilities.

During the TRP testing, battery evolution and temperature profiles are illustrated in Fig. 7b, c. At test initiation, a heating plate was activated, causing gradual temperature escalation in Battery 1. Upon reaching 731 s, its internal temperature attained the critical TR threshold, triggering violent exothermic reactions between electrode materials and electrolyte. Rapid pressure surge activated the safety valve, accompanied by explosive gas venting with visible sparks; following a brief delay, Battery 1 fully entered TR status, with surface temperature T_1,b_ surging to 765.6 °C within 20 s. Absence of an insulating layer permitted direct heat conduction from Battery 1 to adjacent Battery 2, which initiated TR merely 48 s later, peaking at 772.9 °C—exceeding the first TR maximum. Fifty seconds post-Battery 2’s safety valve activation, Battery 3 underwent TR. High-resolution imaging revealed localized melting of the aluminum module casing due to extreme temperatures. Notably, successive TR events exhibited progressively higher peak temperatures, demonstrating cumulative heat accumulation effects. Comparative experiments demonstrated that insulation significantly delays TRP progression. In Test 2 using 3 mm-thick SF insulation, Battery 2’s TR was delayed by 479 s after Battery 1’s TR, while Battery 3’s TR occurred 607 s subsequent to Battery 2’s event, with markedly reduced temperature increments per TR cycle—attributed to both heat barrier effects and dissipative losses introduced by SF. More strikingly, Test 3 employing SF/GFF_APP-ZB-Aero-Kao_ material achieved superior containment: although Battery 2’s frontal temperature T_2,f_ reached 167.1 °C, this surface heating did not elevate its core temperature beyond the TR threshold, successfully confining TR to Battery 1 alone.

Beyond temperature metrics, voltage evolution serves as a critical parameter for monitoring battery TR. As depicted in Fig. 7d, Test 1 exhibited abrupt voltage collapse to 0 V for Battery 1 at 739 s, slightly delayed relative to the timing shown in Fig. 7b. This discrepancy likely arises from sustained violent chemical reactions within the cell during pressure relief valve actuation, ultimately leading to complete TR failure with delayed zero-voltage manifestation. The delay intervals between successive batteries increased progressively (9, 12, and 18 s), reflecting cumulative heat accumulation effects. Similarly, voltage profiles from Tests 2 and 3 demonstrated that enhanced insulation substantially postponed TR onset—Test 2 extended TR initiation to 1,224 and 1,831 s, while Test 3 employing SF/GFF_APP-ZB-Aero-Kao_ successfully suppressed TRP propagation. Voltage oscillations observed in Battery 2 post-Battery 1 TR may stem from moderate temperature elevation altering reaction kinetics, interfacial impedance changes at electrode/electrolyte interfaces, or radiation-induced disruption of electrochemical equilibrium affecting electromotive force.

Concurrently, TR-induced gaseous emissions (H_2_, CO, etc.) accumulate within the confined module space, elevating internal pressure. Pressure fluctuations induce minute deformations in Battery 2’s casing, perturbing electrode/separator alignment and ionic transport pathways, consequently modifying internal resistance and causing voltage fluctuations. Mass loss data (Fig. 7e, f) further corroborated protective efficacy. Test 1’s TRP progression displayed three distinct phases corresponding to sequential TR events in individual cells. Recoil forces during safety valve activation induced characteristic undulations in mass loss curves. Recorded mass losses reached 250.6, 227.8, and 254.2 g for the three cells, with instantaneous loss rates peaking at 324.2, 244.9, and 101.3 g s^−1^, respectively. These variations primarily originated from differences in residual electrolyte content and aluminum enclosure erosion extent among cells. It is noteworthy that under the 3 mm insulation layer condition (Test 2), the total mass loss reached 796.3 g, surpassing Test 1 without insulation. This indicates that the extended TRP duration facilitated more complete internal reactions within the cells. In stark contrast, Test 3 recorded a total mass loss of only 255.4 g, confirming that solely Battery 1 underwent thermal runaway and subsequent combustion. This finding aligns precisely with temperature monitoring data, verifying the high efficacy of the composite in suppressing thermal runaway propagation. To provide a comprehensive comparison, an additional thermal runaway propagation (TRP) test was conducted using a commercial aerogel blanket. Although the commercial aerogel also successfully prevented thermal runaway propagation and exhibited similar voltage fluctuation profiles (Fig. S6a, b), the maximum front surface temperature of Battery 2 T_2,fmax_ reached a slightly higher value of 181.1 °C. Furthermore, the corresponding mass loss results confirm its success in confining the thermal runaway to a single cell (Fig. S6c, d). Therefore, compared to conventional commercial aerogel blankets widely available on the market, our developed composite material demonstrates highly comparable and competitive thermal protection performance for battery systems.

The observed TRP suppression is fundamentally rooted in the material’s structural and thermal properties. This phenomenon originates from the material’s superior thermal insulation capability, which substantially extends the heat transfer path between battery cells and markedly prolongs the required time for calorific transmission. Consequently, only a minimal fraction of heat generated by Cell 1 propagates to Cell 2, effectively retarding and confining the temperature escalation of adjacent units, which subsequently dissipates gradually through combined convective cooling and conductive pathways. Notably, upon reaching elevated temperatures, a robust ceramic layer forms on the surface of the insulating matrix. This ceramicized barrier exhibits dual functionalities: It resists flame propagation and impedes thermal radiation transfer. This immediate passive-to-active transformation drastically cuts off the transient thermal radiation and convective heat transfer. Furthermore, mitigating TRP requires not just a thermal barrier, but one that forms within the critical protection time window. Battery thermal runaway erupts abruptly, demanding the protective layer to be functional within the first 10–30 s. Correlating with our butane torch and cone calorimetry results, the multiscale synergistic fillers enable near-instantaneous surface ceramicization upon exposure to high-temperature flames. This rapid response speed matches the critical time window of TR venting, ensuring the rapid formation of a continuous ceramic barrier that effectively blocks transient thermal radiation and convective heat transfer before catastrophic heat fluxes reach the adjacent cell.

Beyond thermal insulation, the composite provides crucial mechanical shielding. Crucially, in real-world TR scenarios, activated pressure relief valves expel venting gases that form high-velocity jets accompanied by intense abrasive particulates, dynamic shockwaves, and high-temperature flammables. Conventional porous foams typically fail not due to melting, but through severe mechanical erosion and structural perforation. Herein, the exceptional TRP suppression directly originates from the composite’s reinforced impact and abrasion resistance. As demonstrated in our mechanical evaluations, the material retains 93% of its residual stress after 1,000 cycles and exhibits remarkable tensile toughness. During the violent gas venting phase of Battery 1, the embedded GFF skeleton acts as a robust mechanical firewall, effectively absorbing the kinetic energy of the jet. This structural integrity ensures that the thermal barrier is not blown away or punctured, maintaining a continuous shielding layer against the extreme high-pressure impingement. Additionally, the non-sparking characteristic of the ceramicized barrier eliminates the risk of igniting these gases through self-combustion, thereby significantly mitigating fire hazards.

From a practical integration standpoint, the physical form of the protective material is equally important. The 3 mm ultra-thin profile minimizes the parasitic volume occupied by safety components, thereby preserving the volumetric energy density of the battery module. More importantly, the intrinsic conformability and adhesiveness of the modified PDMS matrix guarantee intimate contact with the aluminum cell casings. This seamless integration eliminates interstitial air gaps, fundamentally reducing interfacial thermal contact resistance. Such conformal contact not only prevents localized hot spots during normal cycling but also ensures the protective layer remains firmly attached without delamination under typical operational vibrations.

## Conclusions

Developing highly safe battery systems is critical for the global energy transition but remains challenged by the "high-temperature–high-pressure" extremes of thermal runaway. This study overcomes this bottleneck by engineering a gradient-laminated ceramifiable silicone foam composite. By integrating a flexible ceramifiable silicone foam with a load-bearing GFF skeleton, the design resolves the inherent trade-off between thermal insulation and impact toughness.

The composite demonstrates exceptional long-term durability under the complex, harsh conditions of daily battery operation. Specifically, its highly stable polysiloxane backbone (Si–O–Si) endows the material with inherent resistance to environmental aging and chemical degradation. Concurrently, its robust thermo-mechanical stability—evidenced by maintaining stable elasticity from –40 to 300 °C and retaining 93% residual stress after 1,000 fatigue cycles—enables it to effectively absorb cyclic cell swelling and mechanical vibrations. This comprehensive sustainability guarantees that the composite maintains its structural integrity as a reliable protective barrier throughout the prolonged lifecycle of energy storage systems. Furthermore, upon exposure to fire, the system undergoes synergistic ceramicization where multiscale interactions between flame retardants and precursors construct a dense ceramic barrier composed of α-Zn_3_(PO_4_)_2_ glassy phases and SiO_2_ frameworks. This transformation significantly suppresses combustion, reducing THR by 54.4% and TSP by 87.9%. Crucially, in practical module testing, this architecture efficiently intercepted high-velocity gas jets and blocked heat transfer pathways, successfully confining thermal runaway to a single cell. This capability effectively eliminates the risk of cascading failures, ensuring the ultimate thermal and structural integrity of the entire battery module.

From a practical application perspective, while the current laboratory-scale preparation involves discrete steps, the primary raw materials are commercially abundant and inexpensive. Furthermore, the gradient-laminated foaming strategy is inherently compatible with industrial continuous roll-to-roll manufacturing processes. By providing a robust structural blueprint that successfully withstands extreme high-pressure thermal shocks, this ceramifiable composite architecture paves a highly viable way for constructing intrinsically safe, next-generation energy storage power stations.

## Supplementary Information

Below is the link to the electronic supplementary material.

Supplementary file1 (DOCX 7085 KB)
