# Supplementary material for: Constructing Intrinsically Safe Lithium-Ion Battery Energy Storage via Gradient-Laminated Ceramifiable Silicone Foams
Source: Nanomicro Lett. 2026 May 24;18:384. doi: 10.1007/s40820-026-02228-2 (PMC13199578; doi:10.1007/s40820-026-02228-2)
Supplement: Supplementary file 1 — Supplementary file1 (DOCX 7085 KB) [file 40820_2026_2228_MOESM1_ESM.docx]

Supporting Information for

**Constructing Intrinsically Safe Lithium-Ion Battery Energy Storage via Gradient-Laminated Ceramifiable Silicone Foams**

Shuilai Qiu^1,2^, Jingyao Xu ^1,2^, Congling Shi ^3^*, Laibin Zhang ^1,2^

^1^ College of Safety and Ocean Engineering, China University of Petroleum-Beijing, 18 Fuxue Road, Beijing 102249, P. R. China

^2^ Key Laboratory of Oil and Gas Safety and Emergency Technology, Ministry of Emergency Management, Beijing 102249, P.R. China

^3^ Beijing Key Lab of MFPTS, China Academy of Safety Science and Technology, Beijing 100012, P.R. China

*Corresponding author. E-mail: [shicl@chinasafety.ac.cn](mailto:shicl@chinasafety.ac.cn) (Congling Shi)

**Supplementary Figures and Tables**


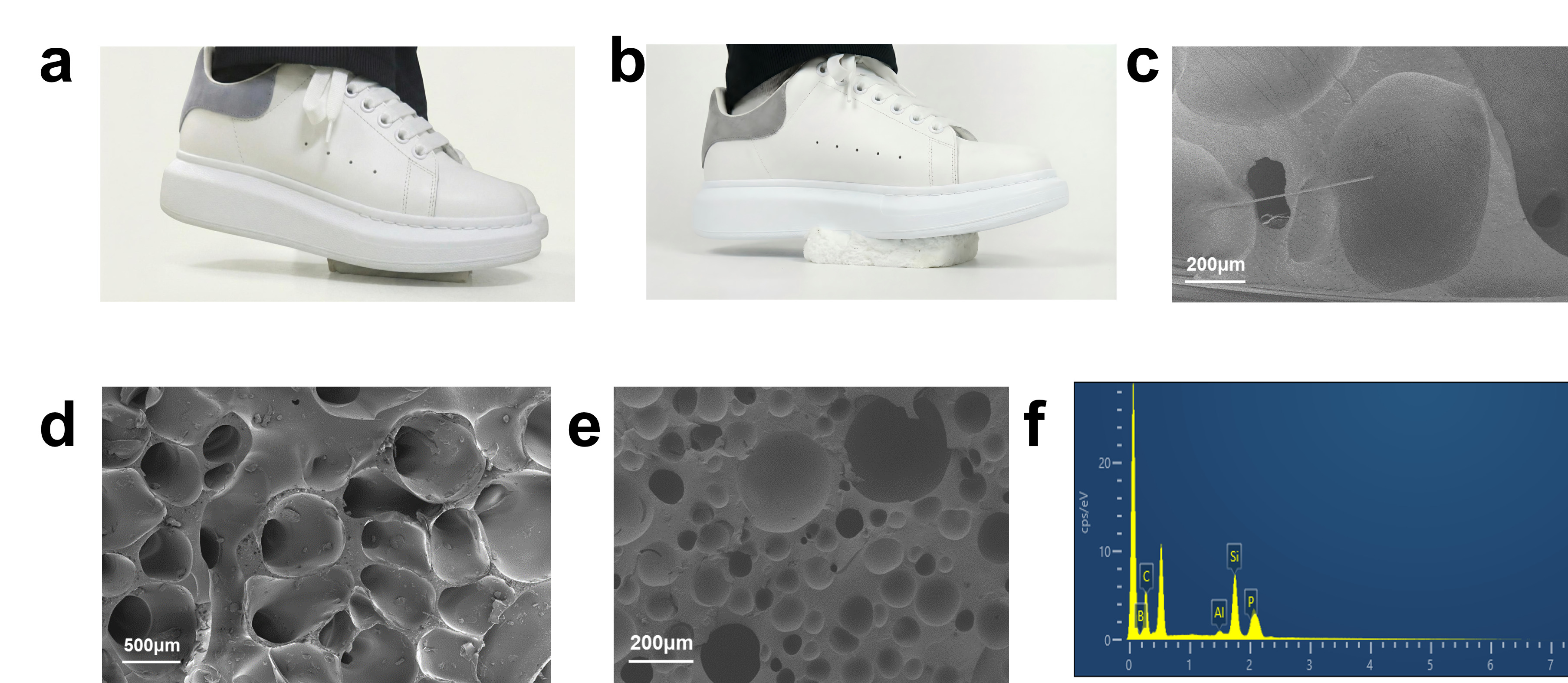


**Fig S1 a, b** Compressive properties of SF/GFF_APP-ZB-Aero-Kao_, **c** Interfacial bonding between SF/GFF_APP-ZB-Aero-Kao_ and glass fiber fabric observed under scanning electron microscopy (SEM), **d** Foam cell structure, **e** Microstructure of SF, **f** Elemental composition distribution within the SF/GFF_APP-ZB-Aero-Kao_.


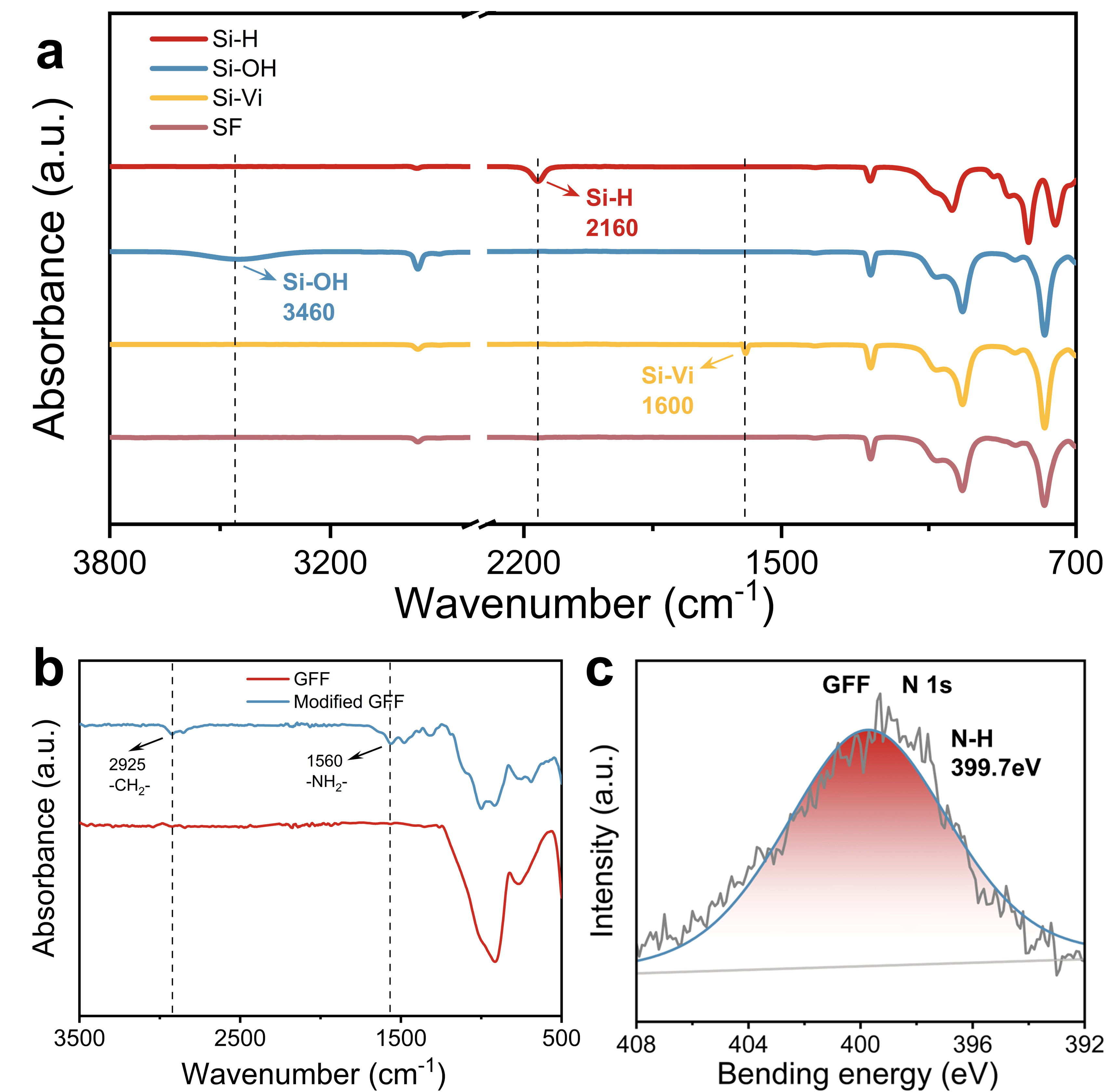


**Fig S2 a** FT-IR spectra of various prepolymers (OH-PDMS, H-PDMS, Vi-PDMS and SF) and **b** FT-IR spectra of GFF and modified GFF**, c** XPS N 1s spectrum of the modified GFF.


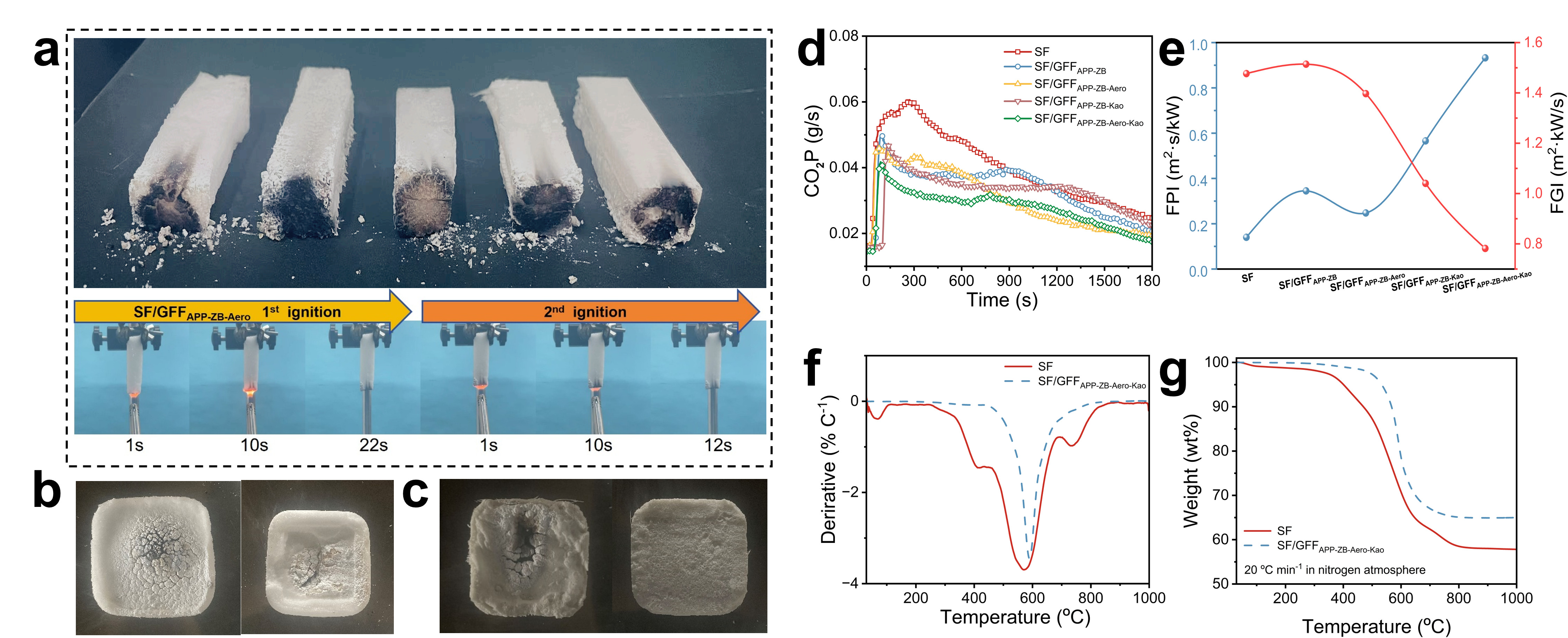


**Fig S3** **a** Char layer morphology at the sample interface post-ignition testing, **b** Front and rear photographs of SF and **c** SF/GFF_APP-ZB-Aero-Kao_ following combustion testing, **d** CO_2_P curves derived from cone calorimetry tests and **e** FPI/FGI performance curves; **f, g** TG analysis curves for SF and SF/GFF_APP-ZB-Aero-Kao_.


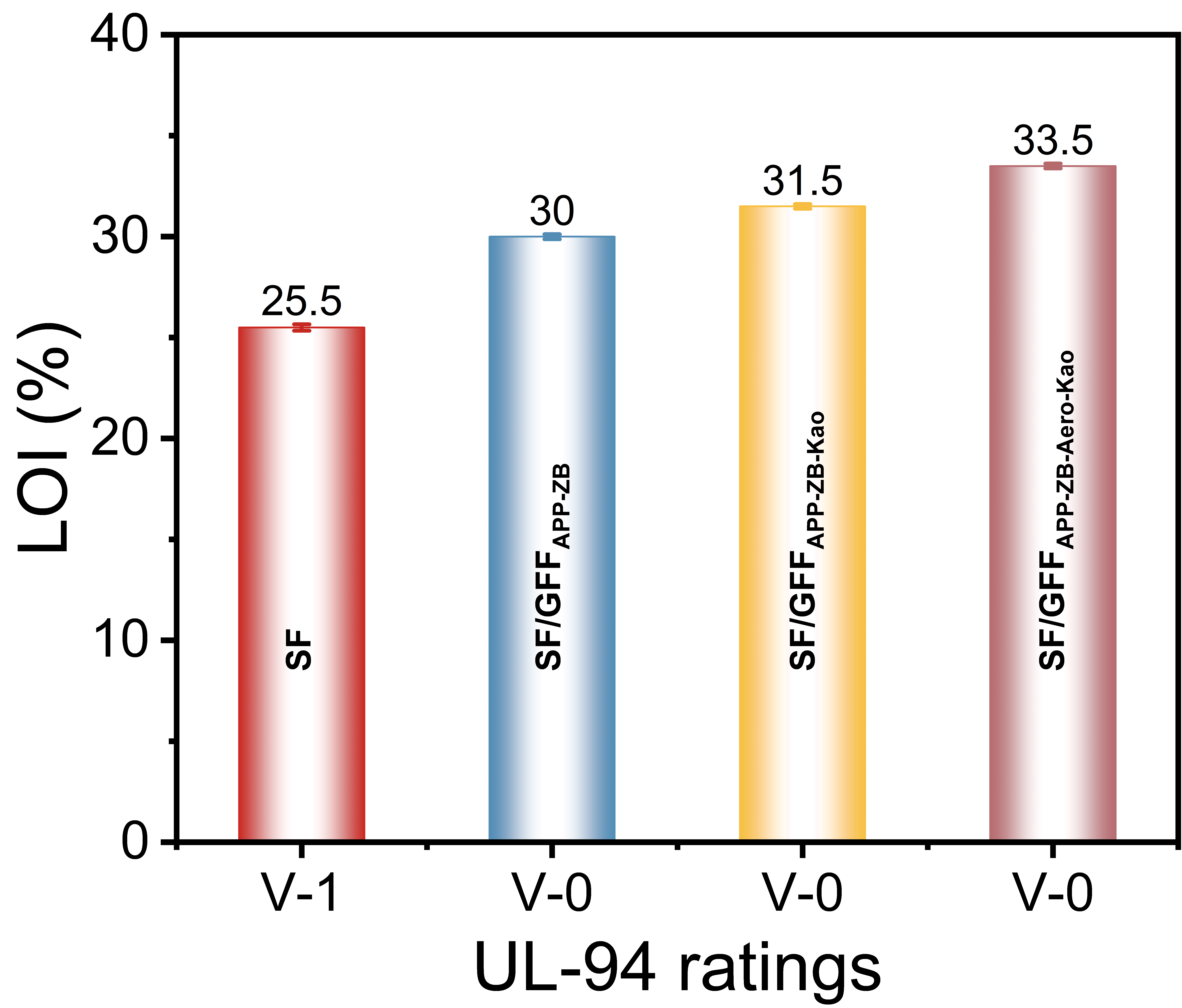


**Fig S4** Fire-resistant properties of SF, SF/GFF_APP-ZB_, SF/GFF_APP-ZB-Kao_, and SF/GFF_APP-ZB-Aero-Kao_.


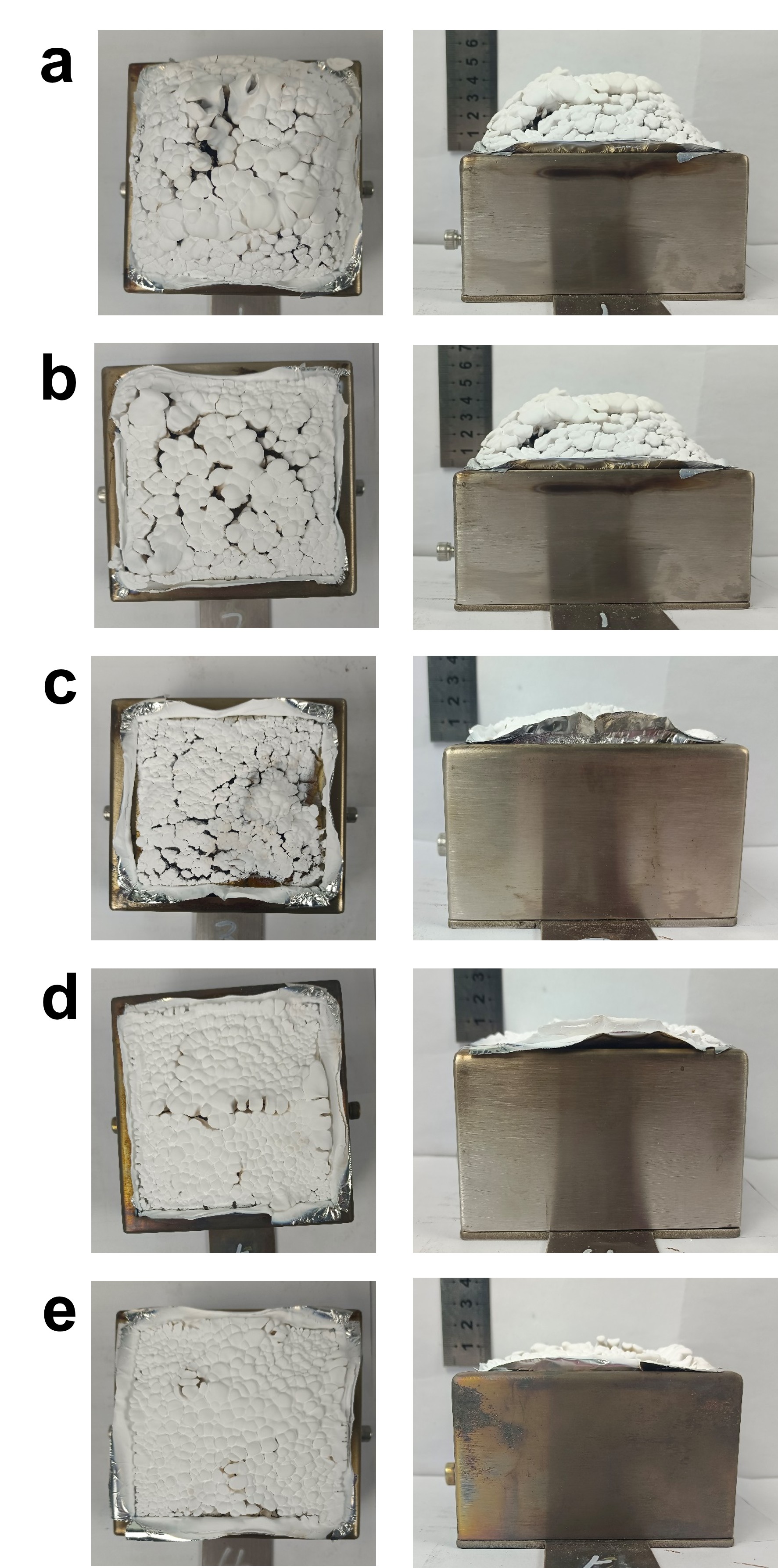


**Fig S5** **a** Post-cone calorimetry testing comparison of SF, **b** SF/GFF_APP-ZB_, **c** SF/GFF_APP-ZB-Aero_, **d** SF/GFF_APP-ZB-Kao_,and **e** SF/GFF_APP-ZB-Aero-Kao_ showing comparative front and side-view images.


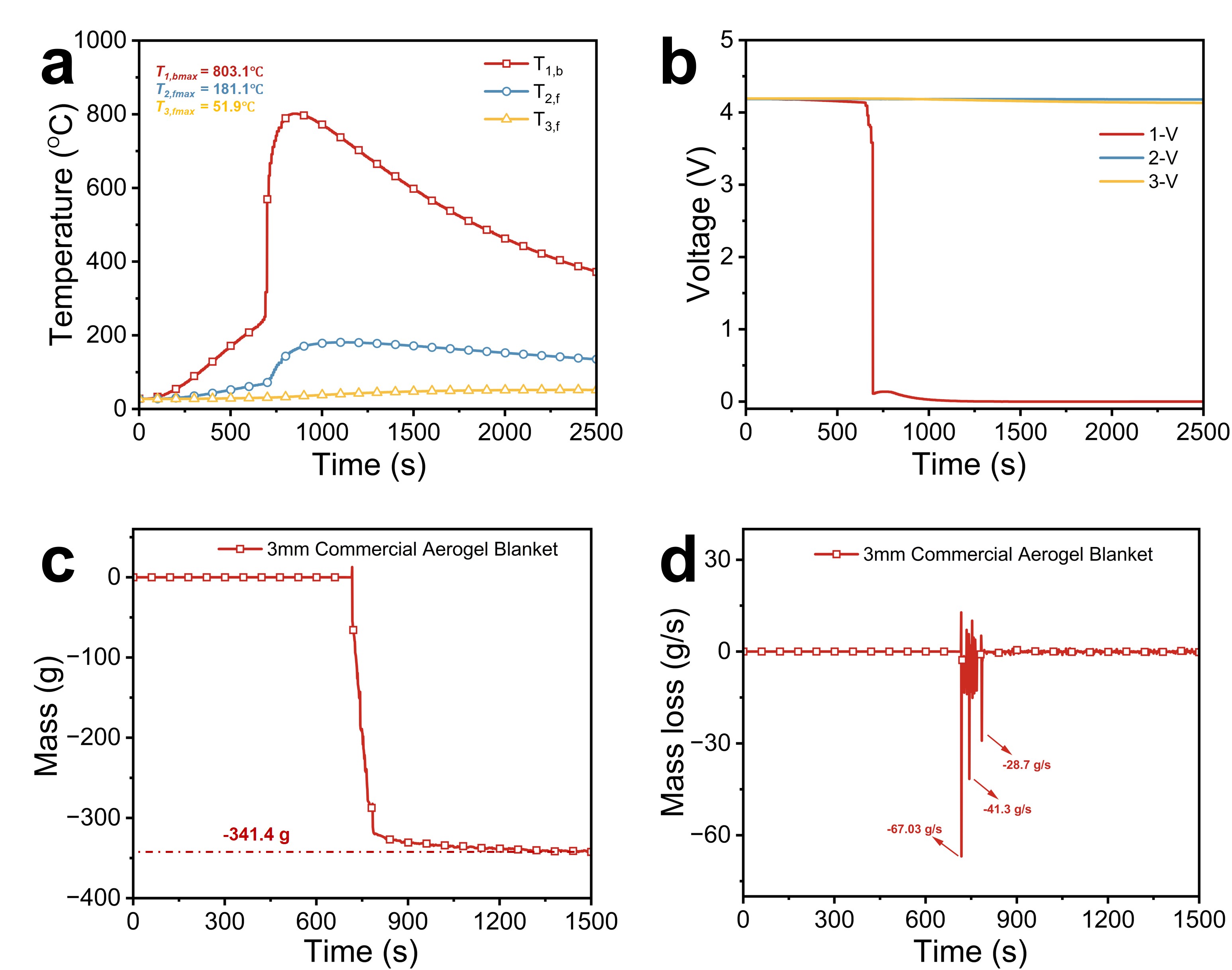


**Fig S6** TRP test results of the battery module using a 3 mm commercial aerogel blanket as the thermal barrier. **a** Temperature evolution curves on the front and rear surfaces of the batteries. **b** Voltage fluctuation profiles during the test. **c** Total mass loss curve. **d** Mass loss rate curve.

**Table S1** Thermal conductivity testing data at 25 ℃.

| Sample code | SF | SF/GFF | SF/GFF_APP-ZB-Aero-Kao_ |
| --- | --- | --- | --- |
| [Thermal conductivity](https://www.sciencedirect.com/topics/chemical-engineering/thermal-conductivity) (W/m·K) | 0.094 | 0.072 | 0.046 |

[Thermal conductivity](https://www.sciencedirect.com/topics/chemical-engineering/thermal-conductivity) of SF, SF/GFF and SF/GFF_APP-ZB-Aero-Kao_ at 25℃, respectively.

**Table S2** Vertical burning test data

| Sample code | SF | SF/GFF_APP-ZB_ | SF/GFF_APP-ZB-Kao_ | SF/GFF_APP-ZB-Aero-Kao_ |
| --- | --- | --- | --- | --- |
| t _1_(s) | 18±3 | 6±1 | 3±1 | 2±1 |
| t_2_ (s) | 5±2 | 4±1 | 2±1 | 1±1 |
| t_1_+t _2_ (s) | 115 | 50 | 25 | 15 |
| t_2_+t _3_(s) | 21±4 | 9±2 | 4±1 | 2±1 |
| Dripping (Y/N) | N | N | N | N |
| UL-94 ratings | V1 | V0 | V0 | V0 |

t_1_ and t_2_ indicate the combustion times of an individual sample after removing the burner.

The aggregate t_1_+ t_2_ refers to the total burning duration for any condition set for the 5 specimens.

t_2_+t_3_ measures the total time of flaming and glowing for a single sample following the second flame application.
